# Decoupled trophic responses to long‐term recovery from acidification and associated browning in lakes

**DOI:** 10.1111/gcb.14580

**Published:** 2019-02-27

**Authors:** Taylor H. Leach, Luke A. Winslow, Nicole M. Hayes, Kevin C. Rose

**Affiliations:** ^1^ Department of Biological Sciences Rensselaer Polytechnic Institute Troy New York; ^2^ Department of Ecology, Evolution and Behavior University of Minnesota St. Paul Minnesota

**Keywords:** acidification, Adirondack Mountains, aquatic, calcium, dissolved organic matter, long‐term trends

## Abstract

Increases in the concentration of dissolved organic matter (DOM) have been documented in many inland waters in recent decades, a process known as “browning”. Previous studies have often used space‐for‐time substitution to examine the direct consequences of increased DOM on lake ecosystems. However, browning often occurs concomitant with other ecologically important water chemistry changes that may interact with or overwhelm any potential ecological response to browning itself. Here we examine a long‐term (~20 year) dataset of 28 lakes in the Adirondack Park, New York, USA, that have undergone strong browning in response to recovery from acidification. With these data, we explored how primary producer and zooplankton consumer populations changed during this time and what physical and chemical changes best predicted these long‐term ecosystem changes. Our results indicate that changes in primary producers are likely driven by reduced water clarity due to browning, independent of changes in nutrients, counter to previously hypothesized primary producer response to browning. In contrast, declines in calcium concomitant with browning play an important role in driving long‐term declines in zooplankton biomass. Our results indicate that responses to browning at different trophic levels are decoupled from one another. Concomitant chemical changes have important implications for our understanding of the response of aquatic ecosystems to browning.

## Graphical abstract

Increases in dissolved organic matter (DOM) have been documented in many inland waters recovering from acidification, a process known as ‘browning’. However, browning occurs concomitant with other ecologically important water chemistry changes that may interact with or overwhelm any potential ecological response to browning. Using a long‐term dataset, we found that changes in the primary producers are likely driven by reduced water clarity due to browning, independent of changes in nutrients, but that declines in calcium play an important role in driving declines in zooplankton biomass. Trophic level responses to acidification‐recovery induced browning are decoupled from one another.

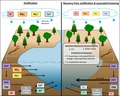

## 1 INTRODUCTION

Dissolved organic matter (DOM), often measured as dissolved organic carbon (DOC) concentration, has increased in recent decades in many inland water bodies across broad regions, including throughout the Northeast USA and Northwestern Europe (Driscoll, Driscoll, Fakhraei, & Civerolo, 2016; Evans, Monteith, & Cooper, 2005; Findlay, 2005; Monteith et al., 2007; Strock, Nelson, Kahl, Saros, & McDowell, 2014; Worrall et al., 2004). Recovery from anthropogenic acidification is considered a primary mechanism driving the increasing DOM of inland waters in most regions (Clark et al., 2010; Driscoll et al., 2016; Monteith et al., 2007), though other broad‐scale changes may dominate or act concurrently in some regions (e.g., precipitation trends (Brothers et al., 2014); terrestrial productivity increases (Finstad et al., 2016); landscape change (Kritzberg, 2017)). These documented increases in DOM, commonly referred to as “browning”, have important ecological implications for aquatic systems. For example, DOM often controls underwater light attenuation (Morris et al., 1995; Williamson, Morris, Pace, & Olson, 1999), thermal structure (Fee, Hecky, Kasian, & Cruikshank, 1996; Read & Rose, 2013; Snucins & Gunn, 2000; Strock, Theodore, Gawley, Ellsworth, & Saros, 2017), and contributes to the role that lakes play in the global carbon cycle (Cole et al., 2007; Drake, Raymond, & Spencer, 2017; Tranvik et al., 2009).

In addition to controlling the physics and biogeochemistry of inland water bodies, studies also indicate that DOM may control aquatic ecosystem productivity (Finstad, Helland, Ugedal, Hesthagen, & Hessen, 2014; Karlsson et al., 2009; Seekell, Lapierre, Ask et al., 2015a; Thrane, Hessen, & Andersen, 2014). Specifically, peak ecosystem productivity may occur at intermediate concentrations of DOM (Finstad et al., 2014; Seekell, Lapierre, Ask et al., 2015a; Solomon et al., 2015). This unimodal relationship between DOM and ecosystem productivity is hypothesized to result from the dominance of a fertilization effect from DOM‐bound nutrients at low DOM concentrations and a shading effect reducing photosynthesis at high DOM concentrations (Solomon et al., 2015). This unimodal hypothesis has been tested and refined using both cross‐sectional (Benoît, Beisner, & Solomon, 2016; Finstad et al., 2014; Kelly, Solomon, Weidel, & Jones, 2014; Seekell, Lapierre, Ask et al., 2015a; Seekell, Lapierre, & Karlsson, 2015b) and experimental studies (Kelly et al., 2016; Koizumi et al., 2018). However, it is currently unclear if this unimodal framework can be used to understand long‐term ecological changes in browning lakes because DOM increases may covary with numerous other environmental changes.

Water chemistry characteristics that correlate with browning through time may be contributing to, or controlling, changes in aquatic ecosystems and overwhelming any potential response to browning itself. For example, in the Adirondack Mountains region of New York, USA, long‐term recovery from acidification has stimulated substantial long‐term browning, altered acid/base chemistry, and modified ion cycling between soil and aquatic ecosystems (Driscoll et al., 2016; Likens, Bormann, & Johnson, 1972). Recovery from soil acidification has reduced aluminum run‐off to sub‐toxic thresholds, allowing extirpated fish populations to recover at some sites (Josephson, Robinson, Chiotti, Jirka, & Kraft, 2014; Lawrence, Dukett, Houck, Snyder, & Capone, 2013; Michelena et al., 2016) and has caused widespread, long‐term declines in biologically important calcium concentrations (Hessen, Andersen, Tominaga, & Finstad, 2017; Jeziorski et al., 2008; Skjelkvåle et al., 2005). The severity of acidification and extent of soil recovery may also control the export of phosphorus from the landscape into inland waters via non‐linear, pH‐dependent processes (Kopáček, Hejzlar, Kaňa, Norton, & Stuchlík, 2015), which in turn may control ecosystem productivity.

To date, joint consideration of the ecological effects of the full suite of water chemistry changes concomitant with browning are rare. To understand past and predict future ecological change in browning waters, it is important to understand the relative importance of increasing DOM versus other co‐occurring physical or chemical changes that may drive long‐term ecological change. Unfortunately few datasets possess the temporal coverage to go beyond space‐for‐time substitution to examine the long‐term consequences associated with browning (but see Williamson et al., 2015), and even fewer have had the comprehensive chemical and biological sampling to evaluate the relative roles of changes in DOM, nutrients, and other dissolved ions in driving long‐term observed ecological change.

**Figure 1:**
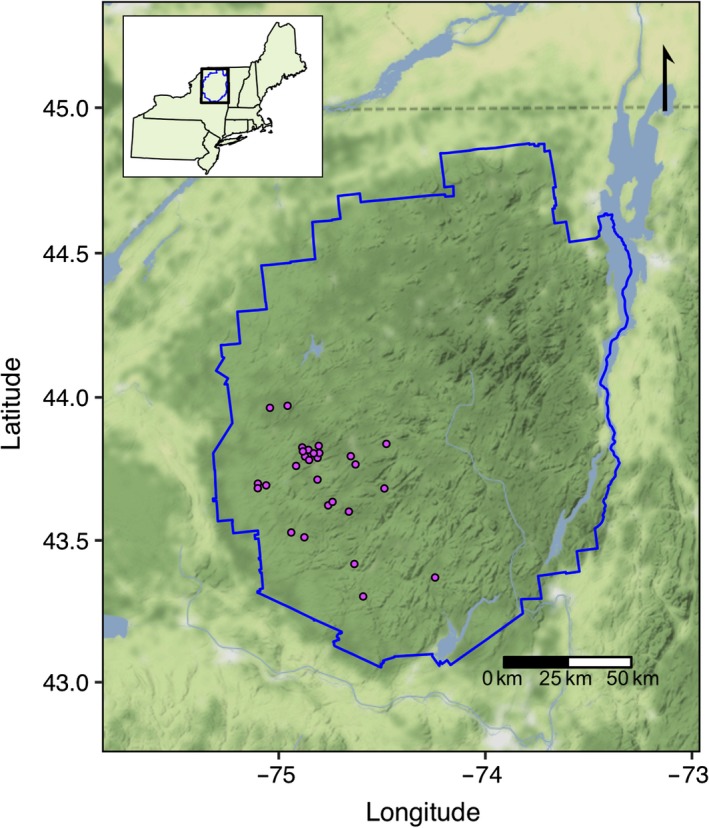
Location of 28 study lakes within the Adirondack State Park (blue line) of northern New York State. Inset shows the location of the park within the northeastern United States

Here, we use a long‐term (19‐year) dataset of physical, chemical, and biological data from 28 lakes in the Adirondack Park, New York, USA (Figure 1) to examine the ecological response of pelagic communities to chemical changes associated with recovery from acidification including browning. These data were collected between 1994 and 2012 from lakes in the Adirondack Park, a region heavily impacted by acid deposition and subsequent recovery, and include a large suite of summertime observations of physical, chemical, and biological variables. Using these data, we examine the following questions: What long‐term changes in primary producer and zooplankton consumer biomass have occurred in these lakes? Are these long‐term ecological changes well predicted by increasing DOC, or do other concomitant physical or water chemistry changes contribute to observed patterns and trends? We compare the relative roles of other ecologically important chemical parameters such as aluminum, calcium, nutrients, and pH versus DOC in explaining long‐term trends in biomass and community composition of planktonic communities. We search for deeper understanding of the temporal scales of coherent change among parameters by examining correlations in both long‐term trends and interannual variability to reduce the chance of spurious relationships between trending datasets. Our results indicate that browning likely contributes to changes in primary producers driven by reduction in water clarity, independent of changes in nutrients but that declining calcium play an important role in driving long‐term change in consumers communities, decoupling long‐term trophic responses to browning.

## 2 MATERIALS AND METHODS

### 2.1 Study site

The long‐term data used in this study are from 28 lakes in the Adirondack Mountains of northern New York State, USA (Figure 1, Table 1). This region received high rates of acid deposition leading up to the 1990 Clean Air Act Amendments (Burns, Lynch, Cosby, Fenn, & Baron, 2011; Driscoll et al., 2001). When combined with inherently low buffering capacity (Jenkins, Roy, Driscoll, & Buerkett, 2005; Omernik & Powers, 1983), these high rates of deposition caused severe acidification of forest soil and surface waters in this region (Driscoll, Newton, Gubala, Baker, & Christensen, 1991; Fakhraei et al., 2014). Because the Adirondack Mountains are within a large, protected State park, land‐use change has been minor.

**Table 1:** Characteristics of the 28 lakes in dataset

Lake	Lat.	Long.	Max. depth (m)	Mean depth (m)	Lake volume (m^3^ × 10^3^)	Surface area (ha)
Big Moose	43.816874	−74.856111	21.3	6.8	34,882	512.5
Brooktrout	43.600966	−74.660624	23.2	8.4	2,420	28.7
Carry^a^	43.682037	−74.488558	4.6	2.2	62	2.8
Cascade	43.789104	−74.812042	6.1	4.2	1719	40.4
Constable^a^	43.831008	−74.806420	4.0	2.1	435	20.6
Dart	43.793758	−74.872572	17.7	7.3	3,807	51.8
G	43.417142	−74.633945	9.8	4.5	1,437	32.2
Grass^a^	43.693004	−75.060844	5.2	1.5	78	5.3
Indian	43.622864	−74.760748	10.7	3.0	981	33.2
Jockeybush	43.302775	−74.591444	11.3	4.5	786	17.3
Limekiln	43.713005	−74.812459	21.9	6.1	11,476	186.9
Long^a^	43.837892	−74.479025	4.0	2.0	33	1.7
Loon Hollow^a^	43.963601	−75.042530	11.6	3.4	191	5.7
Middle Branch^a^	43.699117	−75.100869	5.2	2.1	363	17.0
Middle Settlement^a^	43.682807	−75.101427	11.0	3.4	545	15.8
Moss	43.781396	−74.852986	15.2	5.7	2,598	45.7
North	43.527752	−74.939567	17.7	5.7	10,107	176.8
Queer^a^	43.805956	−74.803521	21.3	10.9	5,960	54.5
Raquette^a^	43.794924	−74.651303	3.0	1.6	24	1.5
Rondaxe	43.760879	−74.915920	10.1	3.0	2,733	90.5
Sagamore	43.766050	−74.628371	22.9	10.5	7,131	68
South	43.510956	−74.875888	18.3	8.3	16,302	197.4
Squash^a^	43.825567	−74.886135	5.8	1.4	45	3.3
Squaw	43.635083	−74.739599	6.7	3.4	1,249	36.4
West^a^	43.811890	−74.882960	5.2	1.5	152	10.4
Willis^a^	43.369628	−74.243171	2.7	1.6	229	14.6
Willys^a^	43.970776	−74.957396	13.7	4.9	1,188	24.3
Windfall^a^	43.804966	−74.830768	6.1	3.2	78	2.4

Latitude (Lat.) and longitude (Long.) are in decimal degrees. All data records start in 1994 and end in either 2006 or 2012.

a Lakes where some data collection ended in 2006.

### 2.2 Data overview

The data analyzed here represent the culmination of work from several different environmental monitoring programs collecting a suite of meteorological, physical, chemical, and biological parameters and an extensive data harmonization effort. Details on data collection, curation, and harmonization are described in Leach, Winslow et al. (2018), though we summarize the collection and curation approach below.

The in‐situ data represent a collation of two independent sampling programs conducted in an overlapping set of lakes. One program focused on water chemistry and the other on biological communities. As a result, not all parameters were collected at the same frequency or on the same day. Variables including mixed layer chlorophyll, phytoplankton, and zooplankton biomass and taxonomy (enumerated to species), nutrients (total nitrogen and phosphorus), iron, and profiles of temperature and dissolved oxygen were collected two times per summer (typically in July and August) from 1994 to 2006 for half of the lakes and from 1994 to 2012 for the remaining half of the lakes (Table 1). All other chemical and meteorological variables (described below) were sampled from 1994 to 2012 for all 28 lakes at a monthly or daily frequency, respectively. To harmonize the mismatched data, we used a ±10‐day sampling window from the collection of the biological data to match samples for all analyses. Data falling beyond the ±10‐day window were not considered combined as a concurrent “sampling event”. This harmonization produced 1,048 unique sampling events with a full suite of physical, chemical, biological, and meteorological measurements (described below).

### 2.3 Physical data

Temperature and dissolved oxygen profiles were collected near the deepest spot in each lake at 1‐meter intervals from the surface to 1 m above the lake bottom. Sampling date varied but profiles were typically measured in July and August. From these profiles we estimated thermocline depth as the seasonal thermocline using *rLakeAnalyzer* (Read et al., 2011). We estimated surface‐ and deep‐water temperature as the average temperature from 0 to 1 m and 1 m above the lake bottom, respectively. Surface and bottom water DO concentration were calculated at the same depths as water temperature. Secchi disk depth was measured at the same time as the temperature and dissolved oxygen profiles.

### 2.4 Meteorological data

To understand how the climate in the region has changed and its potential role in observed ecological change we also include several meteorological parameters in our analyses. Air temperature was extracted from the North American Land Data Simulation (NLDAS; Mitchell, Lohmann, & Houser, 2004) for each lake, and averaged to represent daily values. As an index of the balance between evaporation and precipitation, we used average, monthly estimates of Palmer's drought severity index (PDSI; Alley, 1984) provided by the National Climate Data Center (Vose et al., 2014). The PDSI has previously been shown to be an effective indicator of long‐term climate effects on lake DOM (Jane, Winslow, Remucal, & Rose, 2017).

### 2.5 Chemical data

Total phosphorus (TP), total nitrogen (TN), and iron (Fe) were sampled from integrated epilimnetic samples. For sampling events in unstratified water columns, whole water column integrated samples were used (surface to 1 m above lake bottom). All other water chemistry data represent surface grab samples collected within the top 0.5−1.5 m of each lake. These variables include pH, acid neutralizing capacity (ANC), inorganic monomeric aluminum (Al_IN_), calcium (Ca), magnesium (Mg), potassium (K), DOC, nitrate (NO_3_^‐^), and sulfate (SO_4_^2‐^). Standard methods (EPA, 1987, 1993; Kopp & McKee, 1983; McAvoy, Santore, Shosa, & Driscoll, 1992) were used to measure analytes for all water chemistry and nutrients constituents (see Table 2 in Leach, Winslow et al. (2018). Note that inorganic monomeric Al is calculated as the difference between measured monomeric and organic monomeric Al concentrations.

### 2.6 Biological data

Chlorophyll *a* concentration was analyzed following Turner (1985) from the same integrated, mixed‐layer samples as TP, TN, and Fe (described above). Samples for phytoplankton enumeration were collected with a hose pump over the euphotic zone (determined as 2× the Secchi disk depth). If the euphotic zone extended to the bottom of the lake, phytoplankton samples were collected from the surface to 1 m above the lake bottom. Samples for zooplankton enumeration were collected from the surface to 1 m above the lake bottom or to the depth where DO was <2 mg/L, whichever was shallower. Zooplankton were collected with a continuous flow pump, concentrated with 64 μm mesh, and preserved until analysis. Phytoplankton and zooplankton samples were enumerated and identified to species‐level classifications as outlined in Leach, Winslow et al. (2018). For our analyses here, both phytoplankton and zooplankton data are reported as biomass in mg wet weight L^‐1^ (mg WW L^‐1^), based on species‐specific wet weight parameter conversions (detailed in Leach, Winslow et al. (2018)). For the purposes of our analyses, we examined total zooplankton biomass as well as crustacean zooplankton and rotifer specific biomass as these groups respond differently to acidification (Arnott, Yan, Keller, & Nicholls, 2001; Frost, Fischer, Klug, Arnott, & Montz, 2006). Within the crustacean zooplankton we grouped cladocerans biomass in *Anomopoda*, *Ctenopoda*, and *Gymomera*, and copepods into calanoid or cyclopoid to examine changes in community composition (Table S1). Rotifers biomass was also examined at the genus level (Table S1).

### 2.7 Statistical analyses

#### 2.7.1 Trends

We estimated overall trends in water chemistry parameters, our three ecological responses, and climatic variables. To examine long‐term changes in trophic structure we also estimated trends in community composition (proportion of total community biomass) for crustacean zooplankton and rotifer taxonomic groups (see Table S1). Trends were estimated using a Theil‐Sen slope estimator (referred to here as the Sen's slope; Sen, 1968), which is a non‐parametric trend estimator technique robust to outliers and non‐normality. We estimated the Sen's slope on annual average values across all sites (hereafter, lake population trends; Winslow, Read, Hansen, & Hanson, 2015) and on the annual average values within a lake (hereafter, within lake trends). Since Sen's slope does not include statistical significance, trend significance was assessed with the non‐parametric Mann–Kendall analysis (Yue, Pilon, & Cavadias, 2002).

#### 2.7.2 Correlations in interannual variability (IAV)

**Figure 2:**
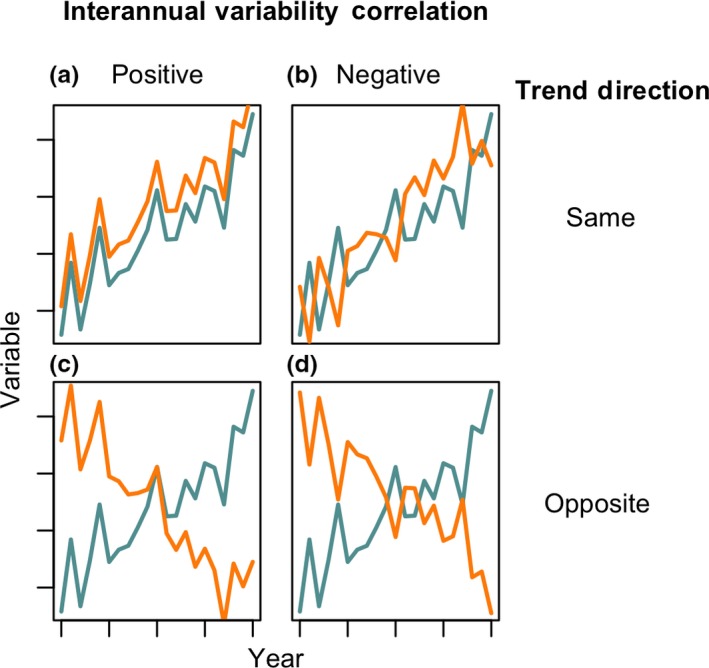
Several, but not all, theoretical combinations of trends and correlation with interannual variability. Interannual variability and trend directions are independent of each other. Directional coherence between long‐term trends are grouped by rows (a and b = same; c and d = opposite) and inter‐annual variability correlations by columns (a and c = positive; b and d = negative). Correlations in interannual variability may also be high even if one, or both, of the variables are not trending over time

It is difficult to infer process from time series data, particularly when multiple parameters are trending over the same time scale. Therefore, in addition to the long‐term trends, we also examined correlations in the first derivatives of each time step in the series within each lake, which allowed us to quantify correlations in the interannual variability (IAV) of any pairwise combinations of variables in our dataset (Figure 2). This analysis is seasonally robust because derivatives were calculated only from seasonally matched samples (i.e., first derivatives were only calculated for July to July or August to August samples across years). Because we could account for seasonality, we used all sampling events rather than annual averages for this analysis. We used a Spearman rank correlation coefficient to quantify the magnitude and significance of these correlations (Best & Roberts, 1975). We chose the Spearman rank correlation coefficient because it assesses monotonic but not necessarily linear relationships between two variables and we had no reason to a priori expect a linear relationship between the IAV of different parameters. Strong positive or negative correlations in IAV can occur independent of the direction or magnitude of trends (Figure 2) and indicate that the yearly movements in the two variables are either mechanistically linked or responding to the same underlying driver.

A substantial challenge in long‐term ecological data analyses is identifying causal drivers of change, particularly when many variables are trending at the same time. Comparisons of both the long‐term trends and the correlations of interannual variability among variables provide one technique to better understand the temporal scales, and possible drivers of coherent change. For example, variables may exhibit both positive long‐term trend correspondence and positive interannual variability correlation, such as bottom‐up stimulation of primary production by DOC‐associated nutrients resulting in positive correlations in both long‐term and inter‐annual variability between DOC, nutrients, and productivity (e.g., Figure 2a). Conversely, correspondence in long‐term trends with inverse interannual variability correlation suggests different mechanisms driving long‐term change and interannual variability (e.g., Figure 2b). For example, summer air temperature and precipitation may both exhibit positive, climatically induced long‐term trends in many regions (Melillo, Richmond, & Yohe, 2014). However, interannual variability between these two variables is often negatively correlated, with wet years tending to having generally cooler temperatures due to increased cloud cover and evaporative cooling (Madden & Williams, 1978; Trenberth & Shea, 2005). Finally, variables may exhibit long‐term trends without any interannual correlation, indicating that there may be some timescales at which the two variables operate independently. When coupled together, long‐term trends and the significance and direction of correlations in IAV between variables can provide a better assessment of potential causality than either test could in isolation.

Our trend and IAV correlation analyses report results from a set of lakes with different temporal extents (13 vs. 19 years). To assess if this biased our trends or IAV correlation estimates, we compared the lake population trends and IAV correlations between the entire dataset, a dataset representing only the subset of lakes with the full 19 years of data, and all data truncated at 2006. We found that all trends and IAV correlations had the same directionality between these three datasets (see Table S2). Some significance was lost with the dataset truncated in 2006, which is not surprising given the smaller dataset and shorter time frame. We therefore report trends and IAV correlations for the entire dataset for all analyses.

All data processing and analyses were performed in the R programming environment (R Developement Core Team, 2015).

## 3 RESULTS

### 3.1 Long‐term trends

**Figure 3:**
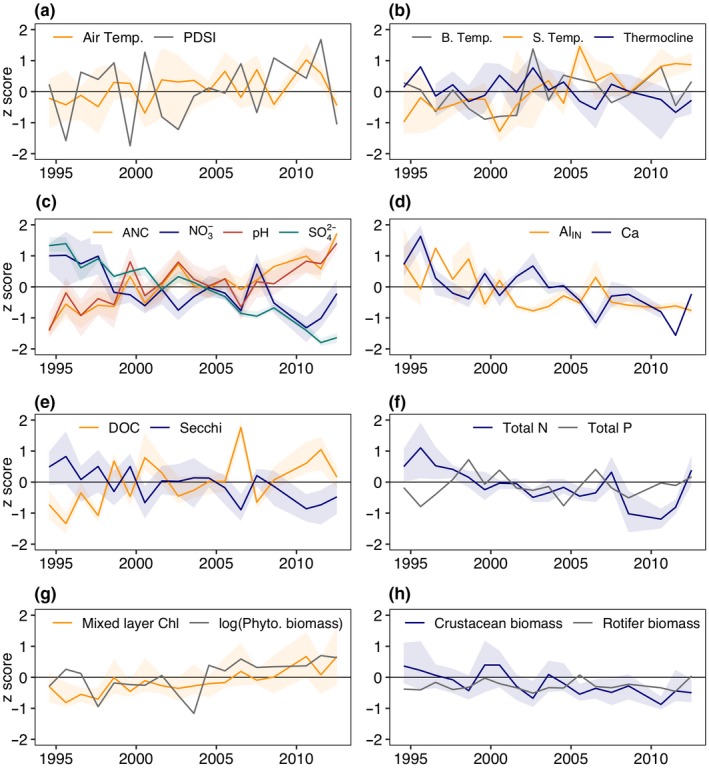
Time series of a) air temperature (0.134 °C year^‐1^) and Palmer drought severity index (PDSI), b) surface (S. Temp; 0.14 °C year^‐1^) and bottom water temperature (B. Temp; no significant trend) and thermocline depth (Thermocline ‐0.04 m year^‐1^), c) metrics of recovery from acidification including pH (0.019 pH year^‐1^), ANC (acid neutralizing capacity; 0.965 μeq. L^‐1^ year^‐1^), and nitrate (NO_3_^‐^; ‐0.023 mg L^‐1^ year^‐1^) and sulfate (SO_4_^2‐^; ‐0.109 mg L^‐1^ year^‐1^) concentrations, d) inorganic monomeric aluminum (Al_IN_; ‐0.89 μg L^‐1^ year^‐1^) and calcium (Ca; ‐0.014 mg L^‐1^ year^‐1^ ) concentration, e) DOC (0.052 mg L^‐1^ year^‐1^) concentration and Secchi disk depth (‐0.046 m year^‐1^), f) total nitrogen (TN; ‐0.009 mg L^‐1^ year^‐1^) and total phosphorus (TP), g) mixed layer chlorophyll concentration (0.06 μg L^‐1^ year^‐1^) and log phytoplankton biomass (Phyto. biomass no significant trend), and h) crustacean (‐0.009 mg wet weight L^‐1^ year^‐1^ and rotifer (no significant trend) biomass. Time series are shown here as a z score (standardized as (value – mean)/ standard deviation) for each variable. Lake population and within lake trends for each variable are reported in Table S3. Lines represent lake population trends as median values for all lakes within a year and shaded areas show the first‐third quartiles of each variable for that year. Lines shown in grey indicate non‐significant trends, while all others represent significant trends (*p* ≤ 0.05) based on a Mann‐Kendall test statistic

Air temperature in the Adirondack region increased at a rate of 0.134°C/year from 1994 to 2012 (*p* = 0.044; Figure 3a). Over this same period, lakes surface temperatures warmed (0.14°C/year; *p* = 0.016) and thermoclines shoaled (−0.04 m/year, *p* = 0.025), but bottom water temperatures did not change (*p* = 0.455; Figure 3b). While there appeared to be cyclic changes in PDSI, there was no overarching long‐term trend (*p* = 0.39).

Lakes showed chemical recovery from acidification with positive trends in pH (0.019 pH units year^‐1^; *p* = 0.002) and ANC (0.966 μeq L^‐1^ year^‐1^; *p* < 0.0001; Figure 3c), and negative trends in SO_4_^2‐^ (−0.109 mg L^‐1^ year^‐1^; *p* < 0.0001) and NO_3_^‐^ concentrations (−0.023 mg NO_3_^‐^ L^‐1^ year^‐1^; *p* = 0.005; Figure 3c). Al_IN_ concentration (−0.89 μg L^‐1^ year^‐1^; *p* = 0.0135; Figure 3d) and concentrations of base cations including Ca (−0.014 mg L^‐1^ year^‐1^; *p* = 0.003; Figure 3d), Mg (−0.003 mg L^‐1^ year^‐1^; *p* = 0. 0,046) and K (−0.0019 mg L^‐1^ year^‐1^; *p* = 0.0365) also declined. Additionally, Fe increased across all lakes (0.005 mg L^‐1^ year^‐1^; *p* < 0.0001). Across the entire population of lakes, DOC concentrations increased (0.052 mg L^‐1^ year^‐1^; *p* = 0.023; Figure 3e) and water clarity declined as indicated by increased water‐color (0.655 Pt‐Co units year^‐1^; *p* = 0.002) as well as shallower Secchi disk measurements through time (−0.046 m/year; *p* = 0.0005; Figure 3e).

Lake population trends showed that chlorophyll concentrations significantly increased (0.060 μg L^‐1^year^‐1^; *p* = 0.0001) and total phytoplankton biomass exhibited a near‐significant positive trend (0.023 log mg WW L^‐1^ year^‐1^; *p* = 0.076; Figure 3g). This trend was not matched by trends in nutrients, with no significant trends in TP or total filterable phosphorus (TFP, which represents dissolved P) across all lakes (*p* = 0.8 and *p* = 0.14, respectively; Figure 3f). TN showed significant negative trends (−0.009 mg L^‐1^ year^‐1^; *p* = 0.0166; Figure 3f) largely driven by declines in NO_3_^‐^.

Zooplankton communities exhibited substantial changes through time. Lake populations trends of crustacean zooplankton biomass declined (−0.009 mg WW L^‐1^ year^‐1^, *p* = 0.0096; Figure 3h) driven largely by declines in calanoid copepod biomass (−0.004 mg WW L^‐1^ year^‐1^; *p* = 0.009), particularly *Leptodiaptomus minutus*, which comprised on average 48% of the crustacean zooplankton biomass in these lakes (first–third Quartile in first and last two years of the data set were 29%–85% and 19%–57%, respectively). Cyclopoid copepods and cladoceran grazers (*Anomopoda* and *Ctenopoda*) did not exhibit trends in biomass through time (*p* = 0.89, 0.96, and 0.98, respectively).

The composition of crustacean zooplankton became less dominated by calanoid copepods (−0.0097 prop mg WW L^‐1^ year^‐1^, *p* = 0.0016) and the community composition shifted, with cladoceran grazers (*Anomopoda*) becoming proportionally more important over time (0.0022 prop. mg WW L^‐1^ year^‐1^; *p* = 0.019). Though because cladoceran grazers did not exhibit trends in biomass through time, the observed increases in cladocerans as a proportion of the total crustacean zooplankton biomass within the community were driven by declines in calanoid copepod biomass, not by an actual increase in cladoceran grazer biomass. Cladoceran grazer (*Anomopoda* and *Ctenopoda*) biomass was generally low, representing on average 24% of total crustacean biomass (first–third Quartile in first and last two years of the data set were 2%–19% and 17%–39%, respectively). Overall rotifer biomass did not show a significant trend (*p* = 0.13). Although the rotifer community became less dominated by *Gastropus* spp. (−0.004 prop. mg WW L^‐1^ year^‐1^; *p* < 0.0001) and *Keratella* spp. (−0.004, *p* = 0.025), no individual rotifer group consistently increased to counter the declines in *Gastropus* and *Keratella* spp.

**Figure 4:**
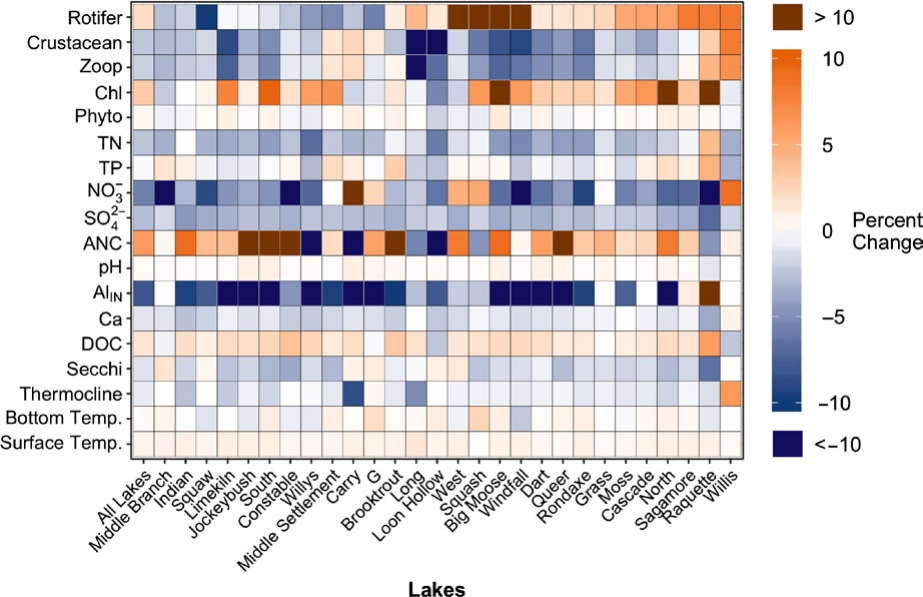
Per cent change in each variable over time for the lake population (all lakes) and each individual lake. Significance of trends is not denoted. Lake population and within lake trends, and per cent change for each variable are reported in Table S3. Rotifer and crustacean represent biomass of each group. All other abbreviations as follows: Al_IN_, inorganic monomeric aluminum; ANC, acid neutralizing capacity; Ca, calcium; Chl, chlorophyll concentration; DOC, dissolved organic carbon; NO_3_^‐^, nitrate; Phyto, phytoplankton biomass; TP, total phosphorus; TN, total nitrogen; Secchi, Secchi disk depth; SO_4_^2^, sulfate; Thermocline, thermocline depth; Temp., temperature; and Zoop, zooplankton biomass

While there were many significant long‐term lake population trends in physical, chemical, and biological characteristics, there was also substantial variability in both the magnitude and direction of within lake trends for some characteristics among the population of lakes (Figure 4). For example, Al_IN_, TN, and SO_4_^2‐^ declined strongly across all lakes, though the concentrations varied, while ANC, TP, and the biological parameters showed higher variability in both the direction and magnitude of within lake trends.

### 3.2 Correspondence among long‐term trends and IAV

**Figure 5:**
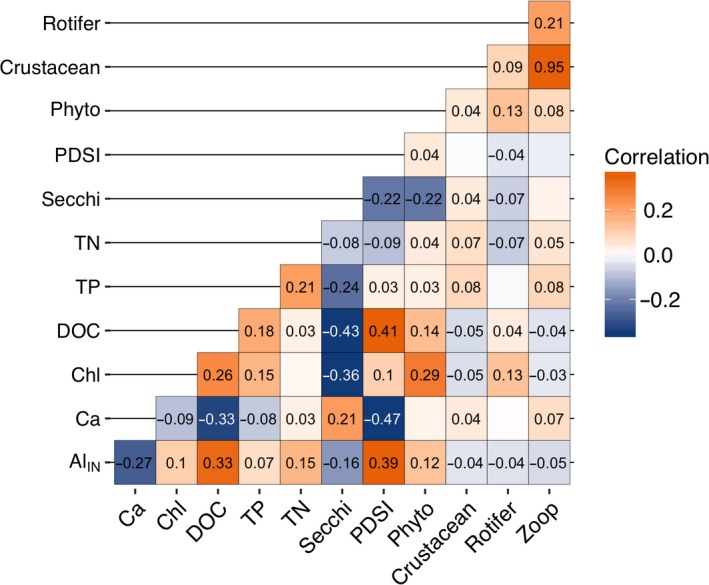
Correlations of interannual variability (IAV) between select variables. Text in each square represents Spearman rank correlation coefficients and squares without values are non‐significant correlations (*p* > 0.01). PDSI is Palmer Drought Severity Index, all other abbreviations as in Figure 4

Within‐lake correlations in IAV exhibited numerous significant correlations, indicating coherence in many chemical and biological variables (Figure 5). We specifically focused on the correlations between potential physical, chemical, and trophic‐mediated drivers of the three ecological measurements: chlorophyll, phytoplankton biomass, and zooplankton biomass. The top three IAV correlates with chlorophyll and phytoplankton biomass were Secchi depth (negative correlation), DOC, and TP (both positive; Spearman coefficient ≥|0.15|, *p* ≤ 0.05; Figure 5). Long‐term trends in chlorophyll and Secchi had opposite directions (chlorophyll increased while Secchi depth declined), which corresponded with the negative IAV correlation between these two variables. Long‐term trends in chlorophyll and DOC were in the same direction (both positive) and also corresponded with their positive IAV correlation. In contrast, chlorophyll and TP showed positive IAV but no correspondence in long‐term trends (TP showed no trend over time, *p* = 0.83).

For zooplankton biomass, the top IAV correlates (ignoring rotifer and crustacean biomass) were phytoplankton biomass, TP, and Ca (all positive correlations; Spearman coefficient ≥0.07, *p* ≤ 0.05; Figure 5) with DOC coming out as negatively, but less correlated interannually (Spearman coefficient = −0.04, *p* ≤ 0.05). Neither TP nor phytoplankton biomass had corresponding significant long‐term trends to match the positive IAV correlation between zooplankton biomass and Ca, which showed long term declines corresponding with the long‐term zooplankton biomass declines. Breaking apart the zooplankton group, Ca was significantly, positively correlated with the IAV of crustacean biomass but not significantly correlated with the IAV of rotifer biomass (*p* > 0.05). The IAV of Al_IN_ was negatively correlated with both crustacean zooplankton and rotifer biomass (both Spearman coefficient = −0.04, *p* ≤ 0.05).

We also examined the relationships between biomass of primary producers and consumers to understand potential trophic‐mediated drivers of change. Chlorophyll and crustacean zooplankton biomass showed opposite long‐term trends and a negative IAV (Spearman coefficient = −0.05, *p* ≤ 0.05), indicating that when zooplankton biomass increased chlorophyll decreased, both seasonally and long term. Meanwhile, phytoplankton biomass showed positive IAV with crustacean zooplankton biomass (Spearman coefficient = 0.04, *p* ≤ 0.05) yet no correspondence in long‐term trends (phytoplankton biomass showed no long‐term trend). Neither rotifer nor phytoplankton biomass showed long‐term trends but showed positive IAV correlation (Spearman coefficient = 0.13, *p* ≤ 0.05).

## 4 DISCUSSION

Our analyses indicate that browning appears to be driving changes in primary producer biomass but that the direct chemical effects of recovery from acidification are overwhelming the consequences of browning for consumer biomass. Long‐term changes in the zooplankton community did not display previously predicted effects of browning despite the increase in primary producer biomass potentially driven by browning. Thus, the long‐term trajectories of these two trophic levels are decoupled from one another. Our results suggest that ecological change in browning lakes may be more dependent upon concomitant environmental changes than previously observed. This has broad implications for assessments of long‐term ecological change associated with browning, given that recovery from acidification, which is driving long‐term changes in our study lakes (Driscoll et al., 2016), is considered a primary driver of increased DOC concentrations in many regions (e.g., Monteith et al., 2007).

Chlorophyll increases, while likely driven by browning, were not due to a fertilization effect from increasing DOM as has been hypothesized by previous studies (e.g., Solomon et al., 2015). Rather, browning‐driven decreases in water clarity and thermocline shoaling may have driven observed increases in mixed layer chlorophyll. Under lower light conditions compensatory increases in chlorophyll can occur as phytoplankton produce more chlorophyll per unit biomass (Fennel & Boss, 2003). This photo adaptation may have contributed to increases in chlorophyll independent of increases in phytoplankton biomass as high DOM and associated low light levels are known to limit primary production (Karlsson et al., 2009; Staehr, Brighenti, Honti, Christensen, & Rose, 2016). Additionally, sub‐epilimnetic peaks in chlorophyll are common when the euphotic zone extends below the thermocline (Brentrup et al., 2016; Leach, Beisner et al., 2018), a condition common in our study lakes. The mixed layer sampling regime used here would have missed chlorophyll below the thermocline, particularly when the thermocline and the euphotic zone are well separated. In situations where the euphotic zone moves closer to the thermocline, such as in our study lakes when the average bottom of the euphotic zone shoaled from 9.3 m to 6.8 m below the thermocline (first two vs. last two study years), some sub‐epilimnetic chlorophyll would likely move into the mixed layer (Brentrup et al., 2016). Sub‐epilimnetic productivity in the suite of stratified study lakes are likely shifting to shallower depths leading to increased epilimnetic integrated chlorophyll without a substantial increase in phytoplankton biomass. Similarly, Carpenter and Pace (2018) also reported increased epilimnetic chlorophyll concentrations with browning waters in a long‐term data set, which they attributed to compression of phytoplankton into a narrower surface layer rather than increases in areal (i.e., whole‐water column) phytoplankton biomass. As an alternative explanation for the increases in chlorophyll, it is possible that the loss of important zooplankton grazers released phytoplankton from top‐down control thereby contributing to increasing chlorophyll trends. Consistent with this hypothesis, long‐term trends in chlorophyll and crustacean biomass were in opposite directions (Figure 3g, h) and the IAV was negatively correlated (Figure 5). However, crustacean biomass was positively correlated on an interannual basis with phytoplankton biomass, suggesting bottom‐up, rather than top‐down trophic interactions.

The long‐term decline in crustacean zooplankton biomass was most likely driven by declining Ca concentrations, not by browning. Our study lakes had long‐term, significant declines in Ca concentration that corresponded with declines in crustacean biomass (Figure 5). Additionally, the interannual variability between these Ca and crustacean biomass was positively correlated (Figure 5), indicating that in years when Ca concentrations were high, crustacean biomass was also high. Declines in surface water Ca concentrations are driven by soil base cation depletion (Driscoll et al., 2001), which has been widely observed in aquatic systems recovering from acidification, and is likely lower today than it was in these lakes before widespread acidification occurred (Hessen et al., 2017; Keller, Dixit, & Heneberry, 2001; Skjelkvåle et al., 2005; Stoddard et al., 1999). Crustacean zooplankton require Ca to build and harden their exoskeletons (Stevenson, 1985) and dissolved ionic Ca in their environment, rather than food, is their main source (Cowgill, 1976). Crustacean zooplankton, particularly *Daphnia*, show reduced reproduction and population growth rates at Ca concentrations <1.5 mg/L (Arnott, Azan, & Ross, 2017; Ashforth & Yan, 2008; Azan & Arnott, 2017). Additionally, a recent series of mesocosm studies showed that calcium levels <1.0 mg/L reduce the population growth rates of several important freshwater copepod species, including *L. minutus *(Arnott et al., 2017), which dominated the crustacean zooplankton biomass in our study lakes. Seven lakes crossed the 1.0 mg/L threshold, and an additional 10 lakes crossed the 1.5 gm/L threshold, by the end of our study period (either 2006 or 2012). Three lakes started below 1.0 mg/L in 1994 but continued to decline and all lakes showed calcium concentrations <3.5 mg/L by the end of the study period.

Previous studies in other regions have linked long‐term declines in *Daphnia *spp. with declining Ca (Jeziorski et al., 2008) and shown shifts in zooplankton community composition towards dominance by less Ca‐demanding daphnid species (Tessier & Horwitz, 1990) or other cladocerans such as *Holopedium* spp. (Hessen, Faafeng, & Andersen, 1995) or *Bosmina *spp. (Azan & Arnott, 2017) across a decreasing Ca gradient. Based on these previous studies we would expect *Daphnia* spp. to suffer most from the observed Ca declines in our study lakes. However, contrary to this we did not detect significant changes in *Daphnia* spp. biomass nor did we observe shifts in the cladoceran composition towards less Ca‐demanding species such as *Holopedium* spp. or *Bosmina* spp. This unexpected result may be due to the fact that cladocerans comprise a relatively small proportion of the overall crustacean zooplankton biomass with many sampling events where *Daphnia* spp., *Holopedium* spp., and/or *Bosmina* spp. were not observed, making trend detection difficult. Cladocerans are considered more sensitive to low Ca than other crustacean zooplankton due to high body Ca content. However, much less is known about the Ca‐demands or thresholds of copepod species as many previous studies have primarily focused on *Daphnia* or other non‐daphnid cladocerans. More recent studies have shown that Ca body content is not always a reliable predictor of species sensitivity to low Ca. (Azan & Arnott, 2017; Azan, Arnott, & Yan, 2015; Tan & Wang, 2010). Thus, the strong relationship between declining Ca and declining copepod biomass observed here suggests that copepods may be more sensitive to low Ca than previously thought, though more research is needed. In contrast with large‐bodied zooplankton, we observed no trends in rotifers (Figure 3h) or correlated interannual variability between rotifers and Ca (Figure 5). Rotifers are generally considered less sensitive to low Ca than crustacean zooplankton (Tessier & Horwitz, 1990) as they have a proteinaceous integument which does not require Ca (Wallace, 2002). Taken together, these results indicate that declining Ca levels likely had a direct negative effect on crustacean zooplankton, but not rotifer zooplankton.

One unexplored factor that may contribute to long‐term declines in crustacean zooplankton is the recovery of fish populations in previously acidified lakes. Soil acidification mobilized Al_IN_ resulting in high concentrations of Al_IN_ in Adirondack lakes and streams which can be toxic to many fish species at concentrations >55 μg/L (Baldigo, Lawrence, & Simonin, 2007; Driscoll et al., 2001). Approximately 40% of study lakes in the first two years of our dataset showed Al_IN_ concentrations above this threshold, but most declined substantially though time (Figure 3d). Recovering fish populations could have increased top‐down predation pressure, thereby causing the observed decline in zooplankton biomass. However, our data and past published research suggest that this is unlikely. Interannual variability in Al_IN_ and zooplankton biomass were not positively correlated, as would be expected from a top‐down aluminum‐mediated increase in fish predation. Additionally, while there has been documented recovery of fish populations in some lakes (Josephson et al., 2014; Sutherland et al., 2015) there is high cross‐lake variability in recovering fish populations, with many Adirondack lakes showing little evidence of fish recovery (Baldigo, Roy, & Driscoll, 2016). While we lack comprehensive time series data to understand if fisheries have recovered in our study lakes, a recent study based on fisheries surveys that included 24 of our 28 study lakes (excluding Big Moose, Cascade, G and South) indicate highly variable recovery, with only four lakes that showed increased total fish biomass >10% and most with no or negative change in fish biomass between approximately 1985 and 2010. Indeed, both the median change in fish species richness and the median change in catch per net night were zero, indicating no or limited changes in fisheries over time. Slow and highly variable recovery of fish populations suggest that changes in fish populations were unlikely a primary factor driving the consistent declines in crustacean zooplankton across lakes.

A positive relationship between in‐lake DOC and P concentrations forms a central assumption of the unimodal hypothesis (i.e., that DOM‐bound nutrients in low DOM, but browning lakes, will fertilize production) and is primarily based on cross‐sectional snap‐shot datasets using space‐for‐time substitution (Kopáček, Hejzlar, Vrba, & Stuchlík, 2011; Seekell, Lapierre, Ask et al., 2015a; Seekell, Lapierre, & Karlsson, 2015b; Thrane et al., 2014). Predictions of how browning lakes will change over time are thus based on the assumption that DOM is an important source of limiting nutrients (Kissman, Williamson, Rose, & Saros, 2013; Solomon et al., 2015) and that DOC and P both increase over time in browning lakes. However, in our study lakes, there were no significant increases in total or filterable P over time despite increases in DOC. Indeed, lakes with positive chlorophyll (11 of 28 lakes) or phytoplankton biomass trends (5 of 28 lakes) all showed either stable or declining TP trends through time (Table S2) which is counter to a fertilization effect. While it is possible that the lack of correspondence between chlorophyll and P trends is because primary producers switched to N limitation or *N*‐P co‐limitation over the course of the data record, such a switch is unlikely due to high TN:TP ratios. Inorganic nitrogen concentrations in our study lakes were high over the entire time period due to nitrogen deposition, and TN:TP molar ratios ranged from 105.2 to 243.2 (first–third quantile), well above the threshold where N limitation typically occurs (22–50 molar ratio; (Guildford & Hecky, 2000; Kolzau et al., 2014). Given the lack of trends in P despite increasing DOC, our results imply there was no fertilization effect from increasing DOM because the DOM is either a poor source of P (nutrient content of DOM has rarely been quantified (Vähätalo, Salonen, Münster, Järvinen, & Wetzel, 2003; Kissman et al., 2013; Daggett, Saros, Lafrancois, Simon, & Amirbahman, 2015), or other concomitant factors are suppressing P increases.

**Figure 6:**
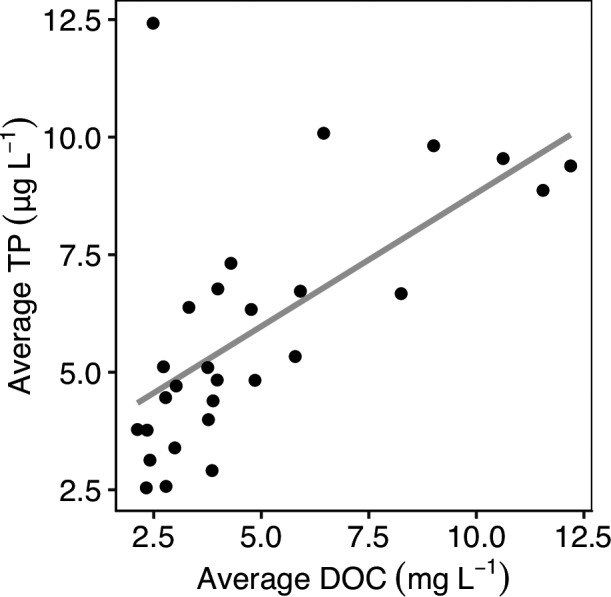
Relationship between DOC and total phosphorus concentrations across all 28 study lakes. Value represents the average concentrations from 1994–2006 or 1994–2012 depending on the lake (Table 1). Line represents a linear model of the relationship with slope 0.57 and intercept 3.1 (R^2^ = 0.39, *p* < 0.001)

Space‐for‐time substitution assumes that long‐term changes in DOM and P export from the terrestrial landscape are correlated analogously to the spatial variability in DOM and P (Kopáček et al., 2015). Despite the lack of correlation in long‐term P and DOC trends in our dataset, among‐lake average DOC and P concentrations were positively correlated (*R^2^* = 0.39, *p* < 0.001; Figure 6). This suggests that the processes that drive DOC and P spatial correlation are different from the processes driving long‐term change in DOC and P. Importantly, the mismatch between spatial and temporal DOC‐P relationships observed here suggest space‐for‐time substitution may yield biased or inaccurate predictions of ecological change in response to long‐term changes in lakes associated with browning.

It is unclear what drives the disconnect in long‐term DOC‐P in our study lakes despite an observed positive correlation in DOC‐P spatially. The disconnect in DOC and P trends may be the result of processes muting P export as we saw no change in P concentrations over time despite increases in DOC. While soil pH has been shown to generally increase P availability (Weil & Brady, 2016), the relationship between soil pH and P sorption capacity of Al and Fe hydroxides may alter the P mobility in soil nonlinearly (Huser, Futter, Wang, & Fölster, 2018; Kopáček et al., 2015). This nonlinearity may alter P availability, and thus export of P to lakes, in complex ways that decouple DOC‐P export. Additionally, legacy N deposition may shift forests toward P limitation (Crowley et al., 2012; Goswami et al., 2018) with higher P retention by terrestrial vegetation (Crowley et al., 2012; See et al., 2015). More research is needed to understand the causes of this disconnect in the long‐term trends in DOC and P.

We did not observe a unimodal pattern of DOC versus our three metrics of ecosystem productivity either across lakes within a year, or in a single lake over time (see Supplement 2). Based on the current understanding of potential drivers of unimodality, this was not entirely unexpected. It has been hypothesized that lake size, which we were unable to control for in our study, alters the DOC concentration at which peak productivity would be observed (Kelly, Solomon, Zwart, & Jones, 2018). The influence of lake size may therefore confound the observation of a cross‐lake unimodality in our data set. Furthermore, while the DOC concentration at peak productivity varies among regions (past work has reported peaks ranging from ~1 mg/L (Finstad et al., 2014) to 15 mg/L (Hanson, Bade, Carpenter, & Kratz, 2003)), the range of DOC concentrations spanned by any single lake over time in our dataset was small compared to the range of DOC concentrations over which unimodal relationships have been observed in other cross‐lake studies (e.g., 1.8–21 mg/L (Seekell, Lapierre, Ask et al., 2015a); ~0.1–100 mg/L (Finstad et al., 2014); 2.2–24.6 mg/L (Hanson et al., 2003)). Lastly, Kelly et al. (2018) hypothesized that DOC and P must be tightly coupled in order to detect a unimodal relationship between DOC and productivity. The lack of coupling between DOC and P over time in our study lakes highlights the limitations of using space‐for‐time substitution in predicting the long‐term ecological implications of browning lakes.

While recovery from acidification has been identified as a primary driver of browning (Clark et al., 2010; Monteith et al., 2007), other drivers such as landscape change (Kritzberg, 2017), climatically induced increases in precipitation (Brothers et al., 2014; Couture, Houle, & Gagnon, 2012; Hongve, Riise, & Kristiansen, 2004), or warming‐induced increases in terrestrial productivity (Finstad et al., 2016; Larsen, Andersen, & Hessen, 2011) and tree line advance (Hofgaard, Tømmervik, Rees, & Hanssen, 2013) may also cause browning in some regions. In these other cases, browning may be a more important driver of ecological change than observed here, because other alternate drivers may have different or muted concomitant water chemistry changes. For example, increased terrestrial productivity or reforestation may increase DOC flux to inland waters, but at the same time increase terrestrial nutrient sequestration, reducing nutrient loading (Huser et al., 2018). Browning caused by increased precipitation or extreme precipitation events may more tightly couple long‐term DOC and nutrient dynamics with or without the accompanying ionic (e.g., Ca and Al_IN_) changes, but may also create more reducing conditions in wetter soils with implications for loading of Fe (Scalenghe, Edwards, Barberis, & Ajmone Marsan, 2010) that can alter light absorbing characteristics of DOC and thus the underwater light environment. Soft water lakes, like our study lakes, were among the most acidified due to low buffering capacity (i.e., low Ca), and have thus exhibited the clearest signals of browning (Monteith et al., 2007). While not all lakes are as soft water as Adirondack lakes, our results, and in particular the importance of declining Ca, may be indicative of other systems undergoing acidification recovery‐induced browning. The degree to which our results are generalizable to regions where browning is occurring independent of recovery from acidification is unknown and highlights the need for integrative long‐term studies that address multiple components of environmental change that often occur simultaneously. However, given that recovery from acidification is considered a primary driver of browning (Monteith et al., 2007), our results are likely generalizable to many other regions.

The drivers of changes in both phytoplankton and zooplankton communities reveal long‐term trends that are decoupled from one another but consistent with the effects of recovery from acidification and in some instances, independent of browning. We found that the chlorophyll increases in Adirondack lakes were likely driven by changing optical conditions associated with browning, but not a fertilization effect predicted by other experimental and cross‐sectional studies. The most likely drivers of zooplankton declines were dominated by variables that change concomitant with increases in DOM, primarily Ca limitation of crustacean zooplankton, not necessarily the direct or trophic‐mediated effects of changing DOM. Lastly, the response of fish population to acidification recovery has a well‐documented link with Al_IN_ toxicity, though recovery has been slower than expected. With different ultimate drivers of browning yielding potentially different concomitant chemistry changes, long‐term ecological changes associated with browning may ultimately depend on the overarching driver causing browning and the interactions of multiple concomitant physical and chemical changes. Our results suggest predicting and managing for the ecological effects of browning is complex in both space and time and requires understanding of trophic level‐specific effects.
