# Supplementary material for: Decoupled trophic responses to long‐term recovery from acidification and associated browning in lakes
Source: Glob Chang Biol. 2019 Feb 27;25(5):1779–92. doi: 10.1111/gcb.14580 (PMC6850094; doi:10.1111/gcb.14580)

Supplemental material

**Decoupled trophic responses to long-term recovery from acidification and associated browning in lakes**

Taylor H. Leach^1^, Luke A. Winslow^1^, Nicole M. Hayes^2^, and Kevin C. Rose^1^

^1^ Rensselaer Polytechnic Institute, Department of Biological Sciences, Troy, NY, 12180 USA

^2^ University of Minnesota, Department of Ecology, Evolution and Behavior, St. Paul, MN, 55108, USA

correspondence: Taylor H. Leach, tel. +1 541 633 9776, email: taylor.leach@gmail.com

Table S1: Taxonomic aggregation used for crustacean and rotifer zooplankton in all data analyses.

| **Group** | **Species** |
| --- | --- |
| ***Crustacean*** |  |
| Anomopoda | *Alona spp., Bosmina longirostris, Bosmina spp., Ceriodaphnia quadrangular, Chydorus spp., Daphnia ambigua, Daphnia catawba, Daphnia dubia, Daphnia mendotae, Daphnia parvula, Daphnia pulex, Daphnia pulicaria, Daphnia retrocurva* |
| Ctenopoda | *Diaphanosoma birgei, Diaphanosoma brachyurum, Holopedium giberum, Sida crystallina* |
| Gymnomera | *Leptodora kindti, Polyphemus pediculus* |
|  |  |
| Calanoid copepod | *Aglaodiaptomus leptpus, Epischura lacustris, Leptodiaptomus minutus, Skistodiaptomus oregonensis* |
| Cyclopoid copepod | *Cyclops scutifer, Mesocyclops edax, Orthocyclops modestus, Tropocyclops extensus, Cyclopoid spp.* |
| ***Rotfier*** |  |
| Ascomorpha | *A. ecaudis, A. ovalis, A. saltans* |
| Collotheca | *C. mutabilis* |
| Conochilus | *C. dossaurius, C. unicornis* |
| Gastropus | *G. stylifer* |
| Kellicottia | *K. bostonensis, K. longispina* |
| Keratella | *K.cochlearis, K. crassa, K. earlinae, K. hiemalis, K. serrulate, K. taurocephala, K. tecta* |
| Ploesoma | *P. truncatum* |
| Polyarthra | *P. euryptera, P. major, P. remata, P. vulgaris* |
| Synchaeta | *S. pectinate* |
| Trichocerca | *T. cyclindrica, T. elongate, T. insignis, T. multicrinis, T. porcellus, T. pusilla, T. rousseleti, Trichocerca spp.* |
| Other | *Anuraeopsis fissa, Anuraeopsis navicular, Asplanchna herricki, Asplanchna priodonta, Beauchampiella eudactylota, Cephalodella gibba, Collurella ucinata, Encentrum spp., Euchlanis pellucida, Filinia terminalis, Hexarthra mira, Lecane crepida, Lecane flexilis, Lecane luna, Lecane mira, Lecane mucronate, Lecane spp., Lecane tenuisetta, Lepadella spp., Microcodon clavus, Monostyla closterocerca, Monostyla copies, Monostyla lunaris, Monostyla obtuse, Trichotria tetractis tetractis* |

**Table S2**: Lake population trends for all lakes in the dataset (overall trend) compared to the lake population trend for the subset of 14 lakes with data record that spans from 1994 – 2012. Trends reported at ns are not significant (at p ≤ 0.05). See Methods for detail on analysis technique. Note that there are not changes in the direction or significance of trends between the full dataset and the subset.

| **Variables** | **Overall trend**  **(unit year ^-1^)** | **Trend for subset of lakes 1994-2012**  **(unit year^-1^)** | **Trend for all lakes 1994-2006**  **(unit year^-1^)** |
| --- | --- | --- | --- |
| Air temperature (°C) | 0.134 | 0.162 | 0.121 (ns) |
| Al_IN_ (μg L^-1^) | -0.89 | -0.92 | -1.488 |
| ANC (μeq. L^-1^) | 0.965 | 1.079 | 1.041 (ns) |
| Bottom temperature (°C) | 0.023 (ns) | 0.019 (ns) | 0.050 (ns) |
| Ca (mg L^-1^) | -0.014 | -0.014 | -0.018 |
| Chlorophyll (μg L^-1^) | 0.06 | 0.07 | 0.061 |
| DOC (mg L^-1^) | 0.052 | 0.052 | 0.083 |
| Fe (mg L^-1^) | 0.005 | 0.005 | 0.008 |
| Phytoplankton biomass (log mg WW L^-1^) | 0.023 | 0.0286 | -0.010 (ns) |
| Mg (mg L^-1^) | -0.003 | -0.003 | -0.003 (ns) |
| Calanoid copepod biomass (mg WW L^-1^) | -0.004 | -0.0057 | -0.009 |
| NO_3_^-^ (mg L^-1^) | -0.023 | -0.034 | -0.026 |
| PDSI (unitless) | 0.119 (ns) | 0.113 (ns) | 0.119 (ns) |
| pH (unitless) | 0.019 | 0.023 | 0.017 (ns) |
| Potassium (mg L^-1^) | -0.0019 | -0.0020 | -0.001(ns) |
| Proportion of biomass Anomopoda (prop. mg WW L^-1^) | 0.0022 | 0.0033 | 0.002 (ns) |
| Proportion of biomass Calanoid (prop. mg WW L^-1^) | -0.0097 | -0.0117 | -0.006 |
| Proportion of biomass Ctenopoda (prop. mg WW L^-1^) | 0 (ns) | 0 (ns) | 0.004 (ns) |
| Proportion of biomass Cyclopoid (prop. mg WW L^-1^) | 0.004 | 0.006 (ns) | 0.003 |
| Proportion of biomass Gastropus (prop. mg WW L^-1^) | -0.004 | -0.004 | 0 (ns) |
| Proportion of biomass Gymnomera (prop. mg WW L^-1^) | 0 (ns) | 0 (ns) | 0 (ns) |
| Proportion of biomass Keratella (prop. mg WW L^-1^) | -0.004 | -0.004 | -0.005 (ns) |
| Proportion of biomass Polyarthra (prop. mg WW L^-1^) | 0 (ns) | -0.004 (ns) | 0.004 (ns) |
| Secchi disk depth (m) | -0.046 | -0.054 | -0.052 |
| SO_4_^2-^ (mg L^-1^) | -0.109 | -0.12 | -0.106 |
| Surface temperature (°C) | 0.14 | 0.168 | 0.081 (ns) |
| Total filterable phosphorus (μg L^-1^) | -0.024 (ns) | -0.019 (ns) | -0.026 (ns) |
| Thermocline depth (m) | -0.04 | -0.047 | -0.030 (ns) |
| Zooplankton: crustacean biomass (mg WW L^-1^) | -0.009 | -0.0128 | -0.013 (ns) |
| Zooplankton: rotifer biomass (mg WW L^-1^) | 0.0004 (ns) | 0.0002 (ns) | 0.882 (ns) |
| Zooplankton: total biomass (mg WW L^-1^) | -0.009 | -0.012 | -0.011 (ns) |
| Total Nitrogen (mg L^-1^) | -0.009 | -0.011 | -0.011 |
| Total Phosphorus (μg L^-1^) | -0.009 (ns) | 0.010 (ns) | -0.015 (ns) |
| Water color (Pt-Co units) | 0.655 | 0.714 | 0.787 |

Table S3: Sen’s slope for each variable for the lake population (All Lakes) and each individual lake. Units are per year. Value in parentheses represents the percent change in each variable calculated at the Sen’s slope divided by the median value of each variable over the data record multiplied by 100. Values in grey represent significant trends using a Mann-Kendall analysis (p < 0.05). Percent values were used to construct Figure 4 in the main text.

| **Lakes** | **Surface Temp. (°C )** | **Bottom Temp.**  **(°C )** | **Thermo-cline**  **depth (m)** | **Secchi Disk Depth (m)** | **DOC**  **(mg L^-1^)** | **Ca**  **(mg L^-1^)** | **Al**  **(μg L^-1^)** | **pH** | **ANC**  **(μeq L ^-1^)** | **SO_4_^2-^**  **(mg L^-1^)** | **NO_3_^-^**  **(mg L^-1^)** | **TP**  **(μg L^-1^)** | **TN**  **(mg L^-1^)** | **Phyto-plankton biomass**  **(log mg WW L^-1^)** | **Chl**  **(μg L^-1^)** | **Zoo-plankton biomass**  **(mg WW L^-1^)** | **Crust-acean biomass**  **(mg WW L^-1^)** | **Rotifer biomass**  **(mg WW L^-1^)** |
| --- | --- | --- | --- | --- | --- | --- | --- | --- | --- | --- | --- | --- | --- | --- | --- | --- | --- | --- |
| All Lakes | 0.14  (0.63) | 0.023  (0.28) | -0.039  (-0.84) | -0.046  (-1.15) | 0.052  (1.44) | -0.014  (-1.02) | -0.897  (-8.25) | 0.019  (0.3) | 0.019  (6.07) | -0.109  (-2.79) | -0.023  (-5.77) | -0.009  (-0.19) | -0.009  (-2.52) | 0.023  (0.42) | 0.060  (3.07) | -0.01  (-1.99) | -0.009  (-2.40) | 0.0004  (2.01) |
| Big Moose | 0.165 (0.75) | 0.063  (0.88) | -0.037  (-0.56) | -0.064  (-1.41) | 0.085  (2.07) | -0.014  (-0.95) | -2.750  (-15.28) | 0.042  (0.71) | 1.209  (9.04) | -0.141  (-3.59) | -0.055  (-6.76) | 0.018  (0.37) | -0.018  (-4.49) | 0.069  (1.26) | 0.182  (10.23) | -0.02  (-7.19) | -0.021  (-8.25) | 0.001  (10.15) |
| Brooktrout | 0.155 (0.71) | 0.013  (0.25) | -0.047  (-0.87) | -0.039  (-0.67) | 0.079  (3.17) | -0.021  (-2.02) | -1.400  (-10.00) | 0.019  (0.33) | 0.686  (10.52) | -0.128  (-3.38) | -0.01  (-2.95) | 0.087  (2.83) | -0.001  (-0.46) | 0.040  (0.71) | 0.036  (1.35) | 0.0005  (0.60) | -0.001  (-2.51) | 0.0002 (0.98) |
| Carry | 0.182 (0.79) | 0.052  (0.24) | -0.212  (-8.66) | 0.029  (0.73) | 0.049  (1.97) | -0.008  (-1.04) | -2.064  (-18.77) | 0.045  (0.91) | 1.428  (-29.21) | -0.091  (-2.76) | 0.003  (10.61) | 0.108  (1.00) | -0.007  (-3.21) | 0.045  (0.86) | -0.034  (-1.77) | 0.033  (2.04) | 0.034  (2.30) | -0.0004 (-2.44) |
| Cascade | 0.154 (0.67) | 0.069  (0.33) | -0.040  (-0.89) | -0.068  (-1.36) | 0.038  (1.26) | -0.011  (-0.49) | -0.167 (0.001) | 0.015  (0.22) | 1.272  (2.39) | -0.101  (-2.15) | -0.041  (-4.06) | 0.039  (0.88) | -0.010  (-2.56) | 0.014  (0.26) | 0.104  (6.43) | -0.016  (-2.14) | -0.029  (-4.00) | 0.001 (5.49) |
| Constable | 0.110 (0.48) | -0.120  (-0.65) | -0.002  (-0.07) | -0.103  (-3.75) | 0.208  (3.61) | -0.026  (-2.08) | -4.750  (-4.70) | 0.010  (0.20) | 0.731  (23.88) | -0.153  (-3.39) | -0.049  (-19.25) | 0.023  (0.43) | -0.010  (-2.56) | -0.018  (-0.35) | 0.032  (1.63) | -0.006  (-0.83) | -0.006  (-0.98) | -0.001  (-2.32) |
| Dart | 0.103 (0.45) | 0.013  (0.19) | -0.028  (-0.52) | -0.025  (-0.58) | 0.069  (1.85) | -0.018  (-1.11) | -1.154  (-10.49) | 0.043  (0.70) | 1.248  (5.87) | -0.136  (-3.44) | -0.053  (-6.44) | -0.013  (-0.30) | -0.013  (-3.33) | 0.035  (0.64) | 0.049  (2.83) | -0.011  (-5.03) | -0.012  (-5.65) | 0.0001 (1.26) |
| G | 0.175 (0.75) | 0.242  (1.86) | 0.019  (0.35) | 0.048  (1.11) | -0.006  (-0.22) | -0.015  (-1.28) | -0.500  (-16.67) | 0.031  (0.50) | 0.926  (5.70) | -0.103  (-2.85) | 0.004  (2.32) | -0.007  (-0.14) | -0.007  (-2.82) | 0.009  (0.16) | -0.029  (-0.98) | -0.006  (-0.90) | 0.007  (1.27) | -0.002  (-5.72) |
| Grass | 0.070 (0.32) | 0  (0) | -0.020  (-0.57) | -0.061  (-1.32) | 0.003  (0.07) | -0.007  (-0.45) | 0  (0) | 0.035  (0.53) | 1.772  (4.50) | -0.075  (-1.69) | 0.0001  (0.11) | 0.014  (0.23) | -0.004  (-1.15) | -0.006  (-0.11) | 0.015  (1.59) | -0.013  (-1.27) | -0.014  (-1.38) | 0.0004  (2.31) |
| Indian | 0.196 (0.86) | -0.013  (-0.16) | -0.092  (-2.60) | -0.043  (-1.71) | 0.110  (1.89) | -0.025  (-2.6) | -4.250  (-9.44) | 0.011  (0.22) | 0.501  (9.12) | -0.153  (-4.55) | -0.001  (-3.13) | 0.06  (0.87) | 0.0002  (0.06) | -0.017  (-0.31) | 0.001  (0.05) | -0.016  (-2.17) | -0.018  (-2.48) | 0.0003  (-1.88) |
| Jockey-bush | 0.191 (0.83) | -0.111  (-0.95) | -0.030  (-0.51) | -0.145  (-1.71) | 0.039  (1.89) | -0.014  (-1.23) | -2.000  (-14.29) | 0.046  (0.82) | 0.892  (12.51) | -0.109  (-2.65) | -0.022  (-3.62) | -0.025  (-0.76) | -0.009  (-3.03) | 0.010  (0.19) | 0.005  (0.83) | -0.015  (-2.67) | -0.019  (-3.31) | 0  (0) |
| Limekiln | 0.207 (0.94) | 0.006  (0.09) | -0.175  (-2.22) | -0.200  (-2.47) | 0.060  (2.02) | -0.011  (-0.61) | -0.271  (-13.54) | 0.016  (0.25) | 1.398  (3.81) | -0.133  (-3.36) | -0.029  (-4.73) | -0.03  (-1.00) | -0.013  (-3.68) | 0.043  (0.81) | 0.082  (7.63) | -0.034  (-7.53) | -0.036  (-8.82) | 0  (0) |
| Long | 0.301 (1.42) | 0.045  (0.35) | -0.079  (-5.23) | -0.004  (-0.24) | 0.208  (1.71) | 0  (0) | -2.514  (-2.59) | 0.005  (0.11) | 0.778  (-5.53) | -0.074  (-2.13) | -0.001  (-2.12) | -0.183  (-2.03) | -0.005  (-1.29) | 0.006  (0.11) | -0.013  (-0.45) | -0.025  (-10.11) | -0.028  (-16.84) | 0.005  (4.17) |
| Loon Hollow | 0.134 (0.61) | 0.054  (0.98) | -0.001  (-0.02) | 0.038  (0.84) | -0.084  (-2.37) | -0.013  (-2.31) | -13.458  (-8.01) | 0.015  (0.31) | 1.131  (-10.62) | -0.065  (-1.81) | -0.032  (-6.33) | -0.073  (-2.52) | -0.021  (-5.96) | -0.097  (-1.85) | -0.073  (-5.45) | -0.007  (-6.72) | -0.006  (-13.22) | 0.001  (1.28) |
| Middle Branch | 0.175 (0.77) | 0.124  (0.66) | 0  (0) | 0.05  (1.56) | -0.022  (-0.51) | -0.019  (-1.09) | 0  (0) | 0.014  (0.20) | 0.294  (0.47) | -0.063  (-1.59) | -0.009  (-23.41) | 0.118  (1.56) | -0.010  (-3.45) | -0.034  (-0.61) | -0.081  (-2.18) | -0.023  (-3.14) | -0.02  (-2.96) | -0.001  (-2.69) |
| Middle Settlement | 0.121 (0.55) | 0.064  (0.87) | -0.042  (-0.90) | -0.188  (-2.98) | 0.032  (1.17) | -0.015  (-1.66) | -1.250  (-9.62) | -0.009  (-0.15) | 0.156  (2.00) | -0.093  (-2.45) | 0  (0) | 0.096  (2.04) | -0.005  (-2.27) | 0.011  (0.20) | 0.068  (6.75) | 0.017  (1.42) | 0.016  (1.49) | -0.001  (-5.25) |
| Moss | 0.108 (0.47) | -0.017  (-0.25) | -0.040  (-0.91) | -0.075  (-1.74) | 0.038  (0.93) | -0.002  (-0.07) | -0.150  (-7.50) | 0.004  (0.06) | 1.627  (1.91) | -0.101  (-2.21) | -0.035  (-5.82) | -0.059  (-1.53) | -0.01  (-3.23) | 0.018  (0.33) | 0.100  (5.24) | -0.003  (-1.00) | -0.006  (-2.53) | 0.001  (5.21) |
| North | 0.188 (0.85) | 0.050  (0.68) | -0.078  (-1.71) | -0.086  (-2.86) | 0.097  (1.99) | -0.015  (-1.16) | -3.759  (-11.39) | 0.035  (0.62) | 1.187  (8.21) | -0.124  (-3.33) | -0.042  (-7.22) | 0.092  (1.85) | -0.008  (-1.74) | 0.049  (0.90) | 0.232  (11.64) | -0.003  (-1.46) | -0.004  (-1.72) | 0.001  (5.52) |
| Queer | 0.083 (0.37) | 0.035  (0.68) | -0.024  (-0.43) | -0.221  (-2.76) | 0.032  (1.16) | -0.017  (-1.05) | -2.00  (-18.18) | 0.039  (0.68) | 1.136  (10.96) | -0.119  (-2.61) | -0.042  (-4.16) | -0.021  (-0.80) | -0.017  (-4.22) | -0.025  (-0.48) | 0.028  (2.55) | -0.019  (-4.44) | -0.018  (-4.52) | 0.0001 (1.43) |
| Raquette | 0.133 (0.69) | -0.111  (-0.92) | -0.018  (-1.31) | -0.110  (-6.50) | 0.583  (5.80) | -0.080  (-3.71) | 3.20  (12.31) | -0.067  (-1.00) | -3.144  (-4.66) | -0.328  (-7.03) | -0.017  (-14.81) | 0.46  (4.59) | 0.023  (4.00) | 0.022  (0.38) | 0.342  (10.52) | 0.017  (4.44) | 0.01  (2.76) | 0.002  (7.94) |
| Rondaxe | 0.186 (0.81) | 0.075  (0.77) | -0.042  (-0.94) | -0.050  (-1.28) | 0.046  (1.29) | -0.011  (-0.55) | -0.188  (-9.38) | 0.014  (0.21) | 2.002  (3.12) | -0.115  (-2.89) | -0.037  (-9.24) | -0.063  (-1.29) | -0.014  (-4.30) | 0.015  (0.28) | 0.063  (2.76) | -0.011  (-5.65) | -0.011  (-6.41) | 0.0002  (1.73) |
| Sagamore | 0.170 (0.80) | 0.021  (0.35) | -0.014  (-0.35) | -0.040  (-1.67) | 0.118  (1.43) | -0.009  (-0.41) | 0.154  (1.10) | 0.019  (0.29) | 1.351  (2.83) | -0.166  (-3.52) | -0.029  (-6.91) | 0.051  (0.74) | -0.002  (-0.42) | 0.038  (0.71) | 0.087  (3.47) | 0.001  (0.46) | -0.001  (-0.30) | 0.001  (8.14) |
| South | 0.194 (0.88) | 0.075  (1.06) | -0.127  (-1.69) | -0.215  (-3.11) | 0.060  (2.41) | -0.011  (-0.87) | -1.967  (-15.13) | 0.052  (0.89) | 1.219  (13.43) | -0.104  (-2.85) | -0.051  (-4.79) | -0.004  (-0.12) | -0.018  (-4.27) | 0.034  (0.63) | 0.162  (9.76) | -0.028  (-5.35) | -0.026  (-5.07) | -0.0003 (-1.06) |
| Squash | 0.033 (0.15) | 0.169  (2.38) | -0.012  (-0.56) | -0.048  (-2.52) | 0.149  (1.67) | -0.002  (-0.34) | -3.356  (-2.40) | 0.009  (0.21) | 1.154  (-4.67) | -0.060  (-1.87) | 0.002  (5.17) | 0.043  (0.40) | -0.002  (-0.45) | -0.041  (-0.77) | 0.167  (6.37) | -0.034  (-4.36) | -0.044  (-6.11) | 0.005  (11.03) |
| Squaw | 0.125 (0.55) | -0.181  (-1.06) | 0.004  (0.08) | 0.017  (0.49) | 0.032  (0.96) | -0.027  (-1.86) | -0.156  (-7.81) | 0.015  (0.24) | 0.84  (3.96) | -0.135  (-3.62) | -0.027  (-8.91) | -0.031  (-0.52) | -0.009  (-3.32) | 0.014  (0.24) | 0.021  (0.60) | -0.019  (-1.76) | -0.017  (-1.60) | -0.002  (-9.93) |
| West | 0.194 (0.85) | -0.075  (-0.54) | -0.012  (-0.49) | 0.023  (1.29) | 0.088  (1.35) | -0.018  (-1.52) | -0.825  (-2.12) | 0.006  (0.12) | 0.938  (8.20) | -0.132  (-3.53) | 0.001  (4.84) | 0.044  (0.42) | -0.006  (-1.29) | -0.033  (-0.59) | -0.081  (-1.83) | -0.011  (-1.04) | -0.018  (-1.70) | 0.007  (15.49) |
| Willis | 0.073 (0.32) | 0.011  (0.05) | 0.083  (6.24) | 0  (0) | -0.263  (-2.36) | 0.017  (0.67) | 0  (0) | 0.004 (0.05) | 0.655  (0.90) | -0.061  (-1.84) | 0.002  (8.96) | -0.271  (-3.25) | -0.011  (-3.50) | -0.023  (-0.38) | -0.033  (-0.83) | 0.045  (6.82) | 0.0340  (8.13) | 0.006  (8.35) |
| Willys | 0.105 (0.49) | -0.083  (-0.86) | -0.017  (-0.27) | -0.117  (-1.46) | 0.057  (2.47) | -0.024  (-2.26) | -20.186  (-12.38) | 0.015 (0.31) | 0.692  (-15.75) | -0.115  (-2.48) | -0.088  (-7.18) | -0.067  (-3.01) | -0.028  (-6.77) | -0.037  (-0.77) | 0.048  (5.88) | -0.002  (-1.08) | -0.004  (-2.26) | -0.001  (-4.53) |
| Windfall | 0.171 (0.77) | -0.336  (-2.30) | -0.036  (-0.80) | -0.050  (-1.35) | 0.095  (2.23) | -0.054  (-1.40) | -0.431  (-14.35) | 0.009 (0.12) | 0.566  (0.51) | -0.139  (-3.02) | -0.064  (-10.13) | -0.153  (-2.40) | -0.023  (-5.33) | -0.026  (-0.47) | 0.226  (6.10) | -0.043  (-6.35) | -0.050  (-9.13) | 0.014  (10.89) |

Supplemental material 2

**DOC vs. chlorophyll, phytoplankton biomass and zooplankton biomass**

While our data set is longitudinal in nature, we subsampled the data set in order to explore spatial patterns among lakes within a year similar to previous space-for-time substitutions (Finstad *et al.*, 2014; Seekell *et al.*, 2015a). Specifically, we compared the relationship between DOC and chlorophyll, phytoplankton and zooplankton biomass, across all lakes within a single year for each year from 1994-2006. This time frame was chosen because those are years where data for all 28 lakes exists, ensuring that we used the most data available and compared across the largest DOC range. Mean annual values of DOC and all three response variables were log transformed to improve normality. For each year we fit a linear and a quadratic model:

$y= \beta_{0}+ \beta_{1}x+ \varepsilon$ (Eqn. 1)

$y= \beta_{0}+ \beta_{1}x+\beta_{2}x^{2}+\varepsilon$ (Eqn. 2)

where, *x* is the log annual mean DOC concentration, *y* is the log annual mean chlorophyll concentration, phytoplankton, or zooplankton biomass. β_0_, β_1_ and β_2_ are the model coefficients for the intercept, slope and quadratic term, respectively, and ε is the error. We used Akaike Information Criterion (AIC; Akaike, 1974) values to compare the fits between the linear and quadratic models.

To examine how the relationship between DOC and the three ecosystem responses changed over time within a single lake we again performed a model comparison between the linear (Eqn. 1) and quadratic (Eqn. 2) models but for all years within a lake. In this analysis we only considered lakes where we have data from the full data range (1994-2012; Table 1) so as to use the longest data range available. To account for multiple comparisons of DOC and the three ecological responses, all regression p-values were adjusted to control for the false discovery rate (Benjamini & Hochberg, 1995).

Using individual years (1994-2006, n = 12 of years) to assess the relationship between DOC concentration and the ecological responses, we found there was a significant positive linear relationship between DOC and chlorophyll across lakes (Fig. S1, Table S4; mean R^2^ = 0.33 ± 0.14, excluding years 2000 and 2001). The only exceptions were 2000 and 2001, where there was no significant relationship. Similarly, there was a positive but non-significant relationship between DOC and phytoplankton biomass for all years considered (mean R^2^ = 0.11 ± 0.05). For both chlorophyll and phytoplankton biomass, a linear model was a better fit than a quadratic model (mean ΔAIC= -1.2 and -1.3, respectively). DOC concentration and zooplankton biomass were negatively correlated, although not significantly (except in 1995), across all lakes within any of the years considered (Fig. S1, Table S4; mean R^2^ = 0.04). There was little consistency across years in whether the linear vs. the quadratic model was a better fit to the zooplankton-DOC data, though given the low explanatory power it is clear that neither fit the data well.

Through time using individual lake time-series, we found a positive, linear relationship between DOC and chlorophyll for lakes with the full time series (1994-2012) with the linear model almost always a better model than the quadratic model (mean ΔAIC= -0.9). We also found that DOC concentration was generally positively correlated with phytoplankton biomass and negatively correlated with zooplankton biomass. However, with few exceptions the relationships between DOC and phytoplankton or zooplankton biomass were not significant (Fig. S2, Table S5).

**Table S4**: Model comparison and coefficients for test of unimodal relationship between DOC and chlorophyll, phytoplankton biomass, and zooplankton biomass across all lakes within each year. Only years with data records that included all 28 lakes were considered. Coefficients are defined in Eqn.1 and Eqn. 2 in the methods and ∆AIC represents the change in Akaike information criteria between the linear and quadratic models. A negative ∆AIC indicates that a linear model described the relationship better than a quadratic model. * indicates p-value ≤ 0.05.

|  | **Linear Model** | | |  | **Quadratic Model** | | | |  |
| --- | --- | --- | --- | --- | --- | --- | --- | --- | --- |
| **Year** | **β_0_** | **β_1_** | **R^2^** |  | **β_0_** | **β_1_** | **β_2_** | **R^2^** | **∆AIC** |
| ***Chlorophyll*** | | | | | | | | | |
| 1994 | -0.16 | 0.86 | 0.48* |  | -0.35 | 1.65 | -0.67 | 0.53* | 0.41 |
| 1995 | -0.14 | 0.69 | 0.28* |  | -0.17 | 0.89 | -0.20 | 0.29* | -1.81 |
| 1996 | -0.19 | 0.67 | 0.26* |  | -0.09 | 0.34 | 0.25 | 0.26 | -1.89 |
| 1997 | -0.13 | 0.59 | 0.18* |  | -0.30 | 1.27 | -0.58 | 0.19 | -1.61 |
| 1998 | 0.06 | 0.44 | 0.18* |  | -0.28 | 1.51 | -0.74 | 0.21 | -0.97 |
| 1999 | -0.17 | 0.72 | 0.40* |  | -0.41 | 1.63 | -0.71 | 0.43* | -0.70 |
| 2000 | 0.06 | 0.40 | 0.06 |  | -0.15 | 1.00 | -0.39 | 0.07 | -1.92 |
| 2001 | -0.03 | 0.47 | 0.12 |  | -0.07 | 0.60 | -0.09 | 0.12 | -1.99 |
| 2002 | -0.01 | 0.49 | 0.17* |  | -0.15 | 0.95 | -0.31 | 0.17 | -1.84 |
| 2003 | -0.21 | 0.92 | 0.48* |  | -0.29 | 1.15 | -0.16 | 0.49* | -1.95 |
| 2004 | -0.18 | 0.78 | 0.48* |  | -0.62 | 2.22 | -1.05 | 0.52* | 0.33 |
| 2005 | -0.14 | 0.81 | 0.49* |  | -0.53 | 2.10 | -0.92 | 0.52* | -0.20 |
| 2006 | 0.05 | 0.51 | 0.20* |  | 0.16 | 0.21 | 0.19 | 0.20 | -1.96 |
| ***Phytoplankton biomass*** | | | | | | | | | |
| 1994 | 5.19 | 0.59 | 0.10 |  | 5.21 | 0.48 | 0.09 | 0.10 | -1.99 |
| 1995 | 5.44 | 0.62 | 0.14 |  | 5.30 | 1.43 | -0.83 | 0.20 | -0.15 |
| 1996 | 5.26 | 0.59 | 0.16 |  | 5.70 | -0.97 | 1.20 | 0.21 | -0.27 |
| 1997 | 4.92 | 0.36 | 0.04 |  | 4.94 | 0.28 | 0.07 | 0.04 | -2.00 |
| 1998 | 4.96 | 0.81 | 0.15 |  | 4.50 | 2.25 | -0.99 | 0.17 | -1.55 |
| 1999 | 4.98 | 0.66 | 0.16 |  | 5.40 | -0.88 | 1.21 | 0.20 | -0.76 |
| 2000 | 4.95 | 0.32 | 0.03 |  | 6.05 | -2.80 | 2.02 | 0.07 | -0.63 |
| 2001 | 5.12 | 0.68 | 0.14 |  | 5.25 | 0.28 | 0.28 | 0.14 | -1.96 |
| 2002 | 4.67 | 0.59 | 0.11 |  | 4.40 | 1.45 | -0.58 | 0.12 | -1.75 |
| 2003 | 4.44 | 0.80 | 0.14 |  | 4.55 | 0.48 | 0.22 | 0.14 | -1.98 |
| 2004 | 5.64 | 0.21 | 0.01 |  | 4.70 | 3.29 | -2.23 | 0.06 | -0.46 |
| 2005 | 5.07 | 0.57 | 0.07 |  | 4.55 | 2.27 | -1.21 | 0.09 | -1.49 |
| 2006 | 5.31 | 0.72 | 0.15 |  | 5.78 | -0.57 | 0.80 | 0.16 | -1.74 |
| ***Zooplankton biomass*** | | | | | | | | | |
| 1994 | -0.23 | 0.02 | <0.01 |  | -0.39 | 0.71 | -0.59 | 0.04 | -0.92 |
| 1995 | -0.51 | 0.67 | 0.14 |  | -0.79 | 2.27 | -1.64 | 0.33* | 4.76 |
| 1996 | -0.28 | 0.08 | <0.01 |  | -0.57 | 1.12 | -0.80 | 0.03 | -1.15 |
| 1997 | -0.51 | 0.43 | 0.07 |  | -0.69 | 1.16 | -0.63 | 0.08 | -1.70 |
| 1998 | -0.31 | -0.23 | 0.06 |  | -0.06 | -1.01 | 0.54 | 0.07 | -1.46 |
| 1999 | -0.32 | 0.27 | 0.03 |  | -0.50 | 0.94 | -0.52 | 0.04 | -1.79 |
| 2000 | 0.04 | -0.35 | 0.03 |  | 1.61 | -4.79 | 2.89 | 0.13 | 0.86 |
| 2001 | -0.57 | 0.37 | 0.08 |  | -0.78 | 1.02 | -0.46 | 0.09 | -1.78 |
| 2002 | -0.71 | 0.39 | 0.06 |  | -0.68 | 0.29 | 0.07 | 0.06 | -2.00 |
| 2003 | -0.20 | -0.24 | 0.01 |  | 0.29 | -1.81 | 1.10 | 0.03 | -1.47 |
| 2004 | -0.34 | 0.10 | <0.01 |  | 0.28 | -1.94 | 1.48 | 0.05 | -0.68 |
| 2005 | -0.44 | 0.09 | <0.01 |  | -0.52 | 0.35 | -0.19 | <0.01 | -1.98 |
| 2006 | -0.44 | 0.10 | <0.01 |  | -2.61 | 5.95 | -3.65 | 0.11 | 1.28 |
|  | | |  |  |  |  |  |  |  |

**Figure S1**: Relationship between DOC and chlorophyll concentration, phytoplankton and zooplankton biomass, across all lakes within a single year. Only 1994-2006 is shown as those are years where data for all 28 lakes exists. All values represent annual means and are plotted in log-log space to better compare with previous studies (Finstad *et al.*, 2014; Seekell *et al.*, 2015).


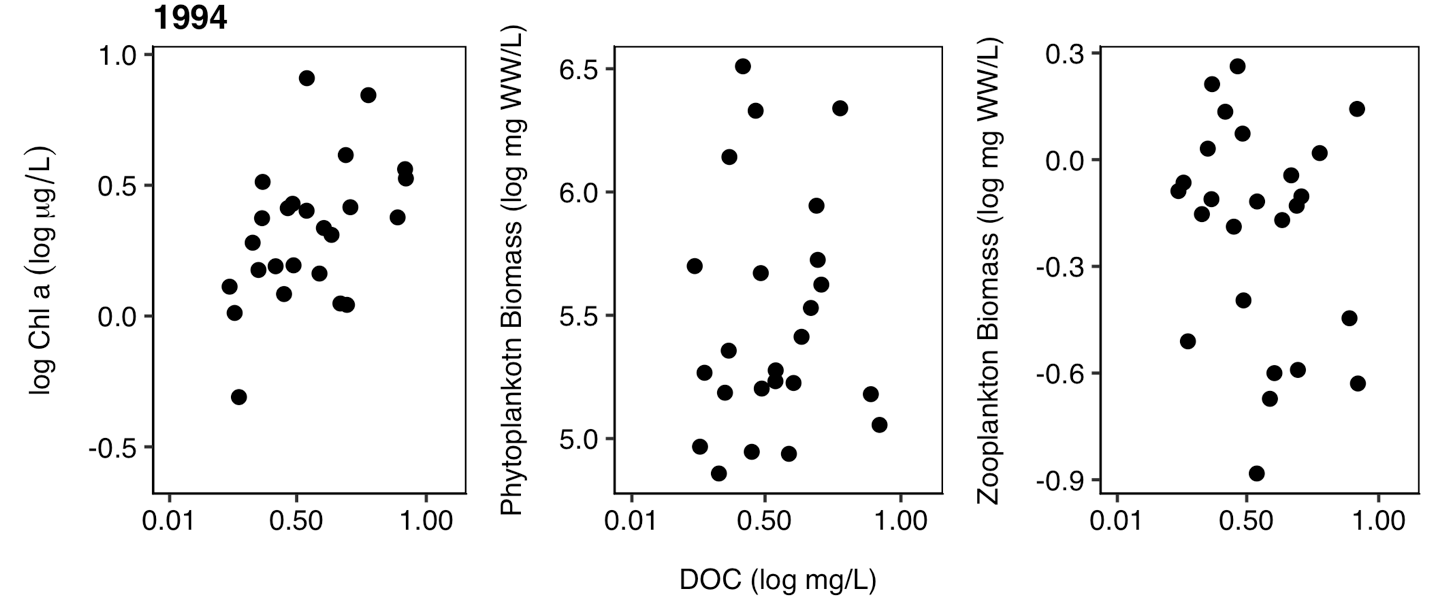

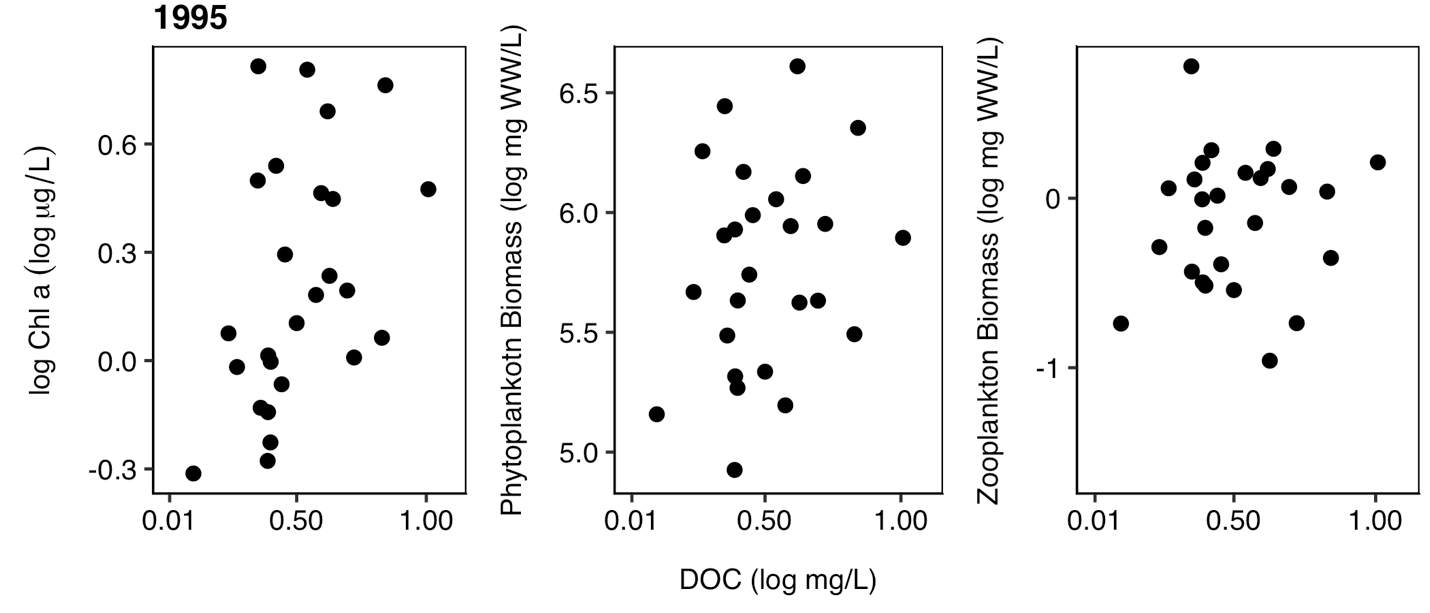

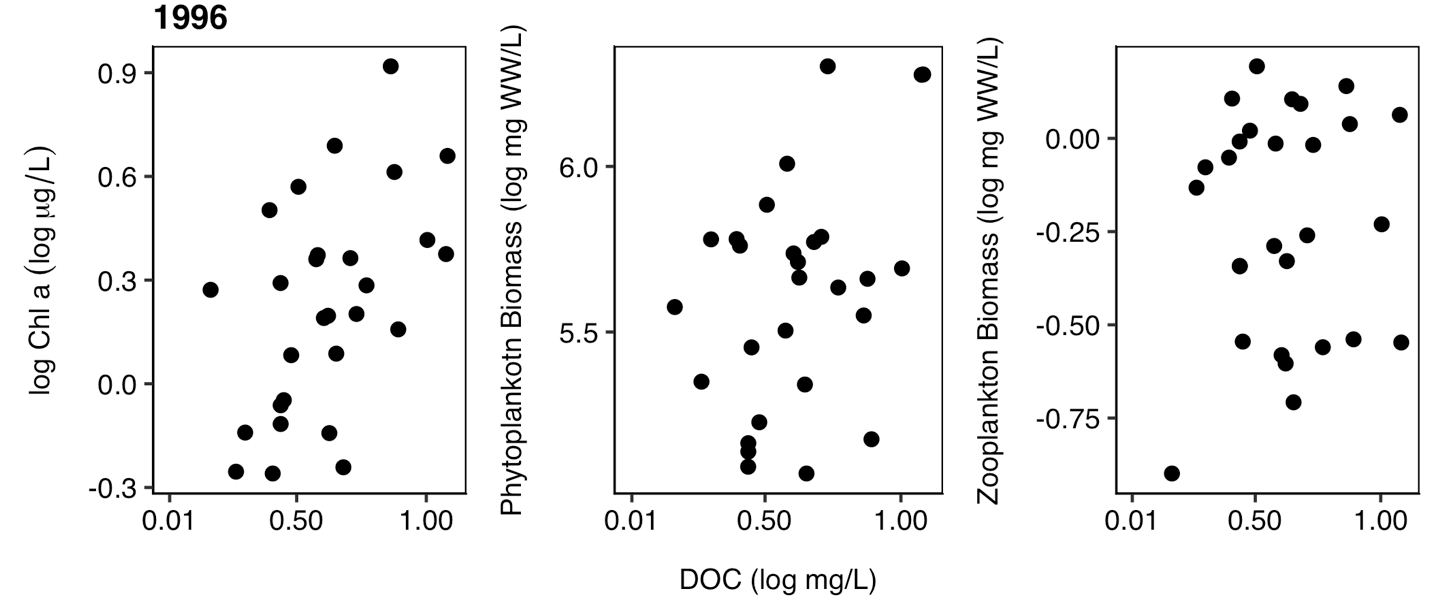

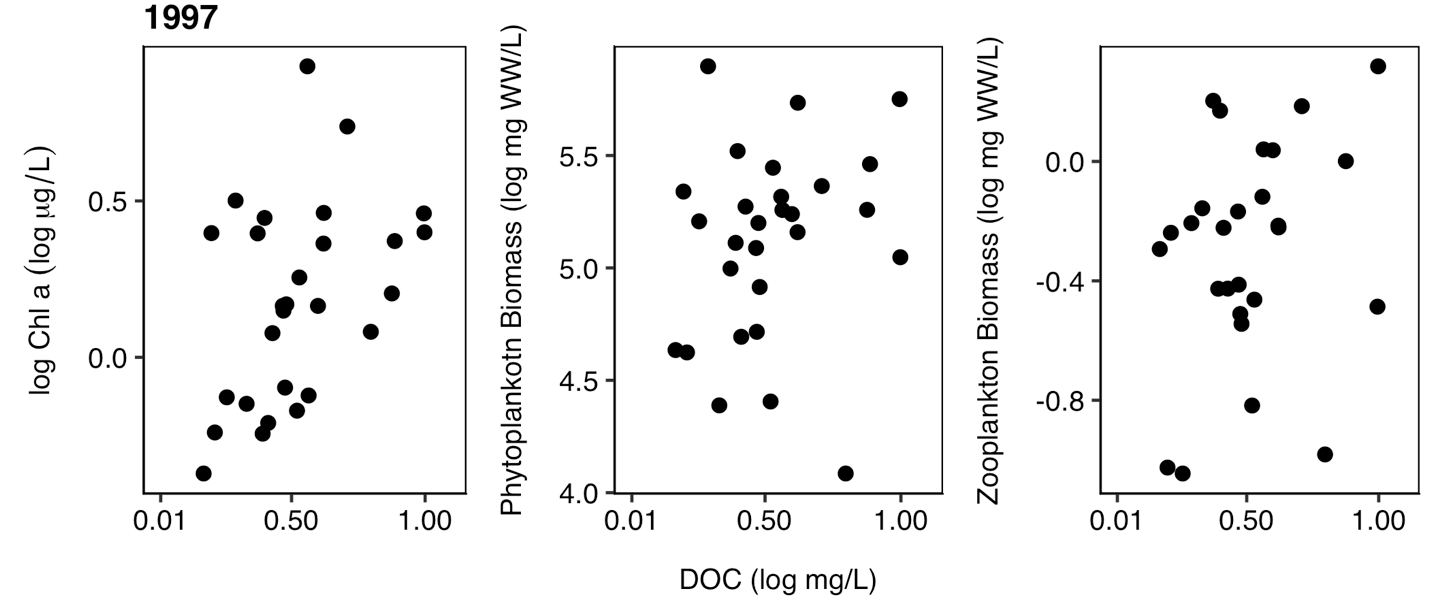

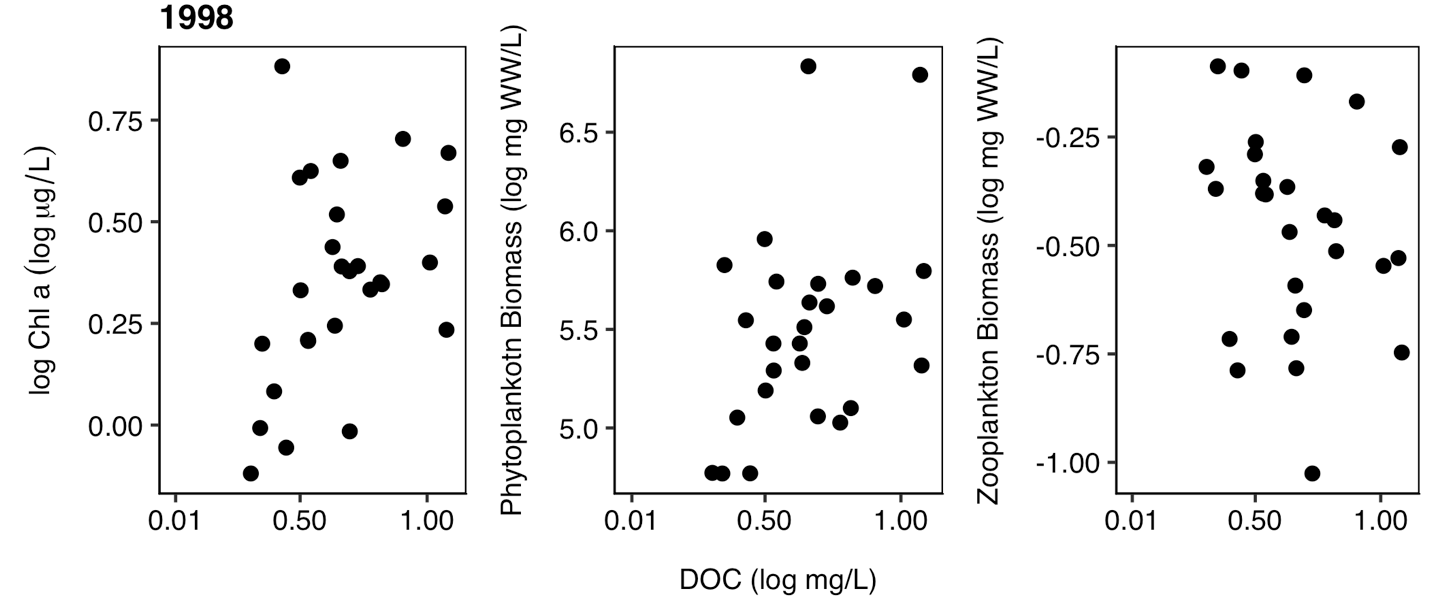

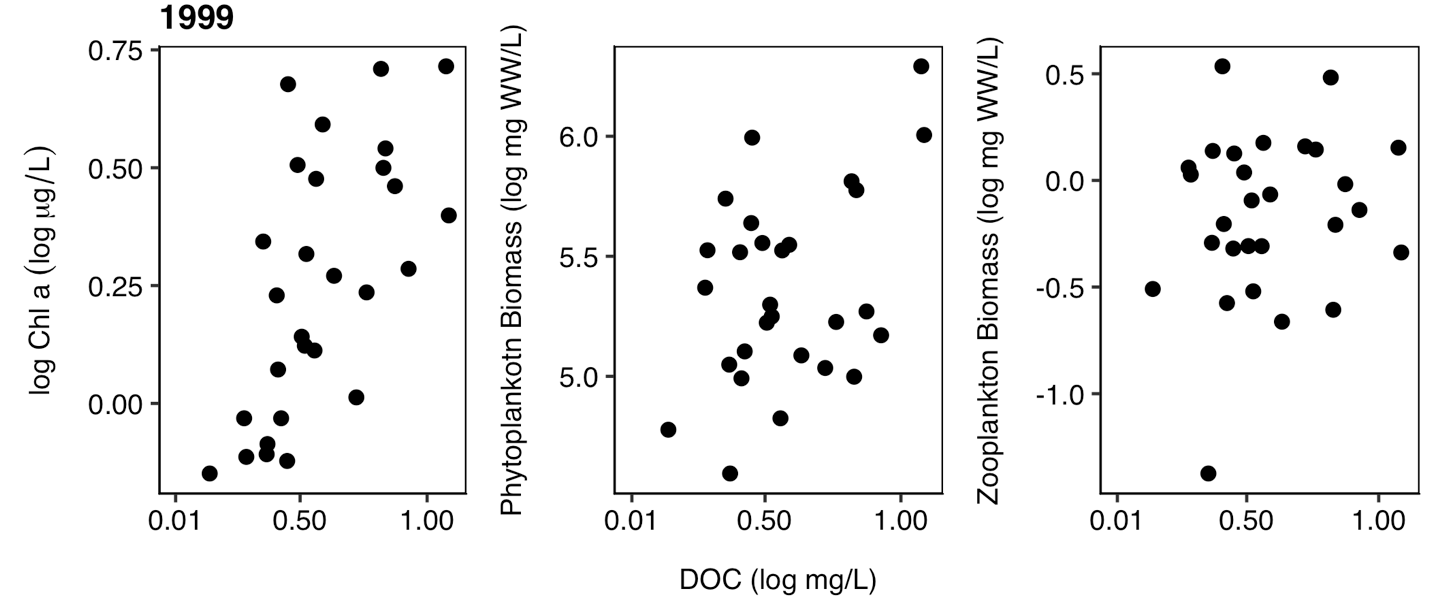

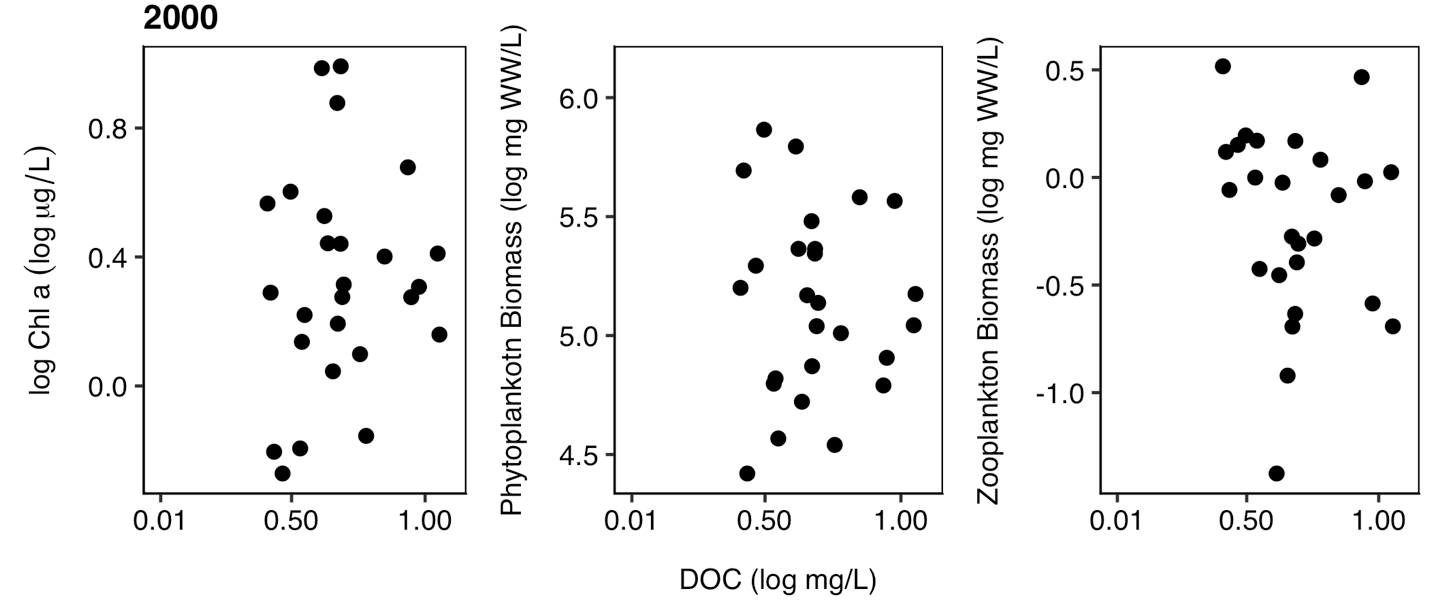

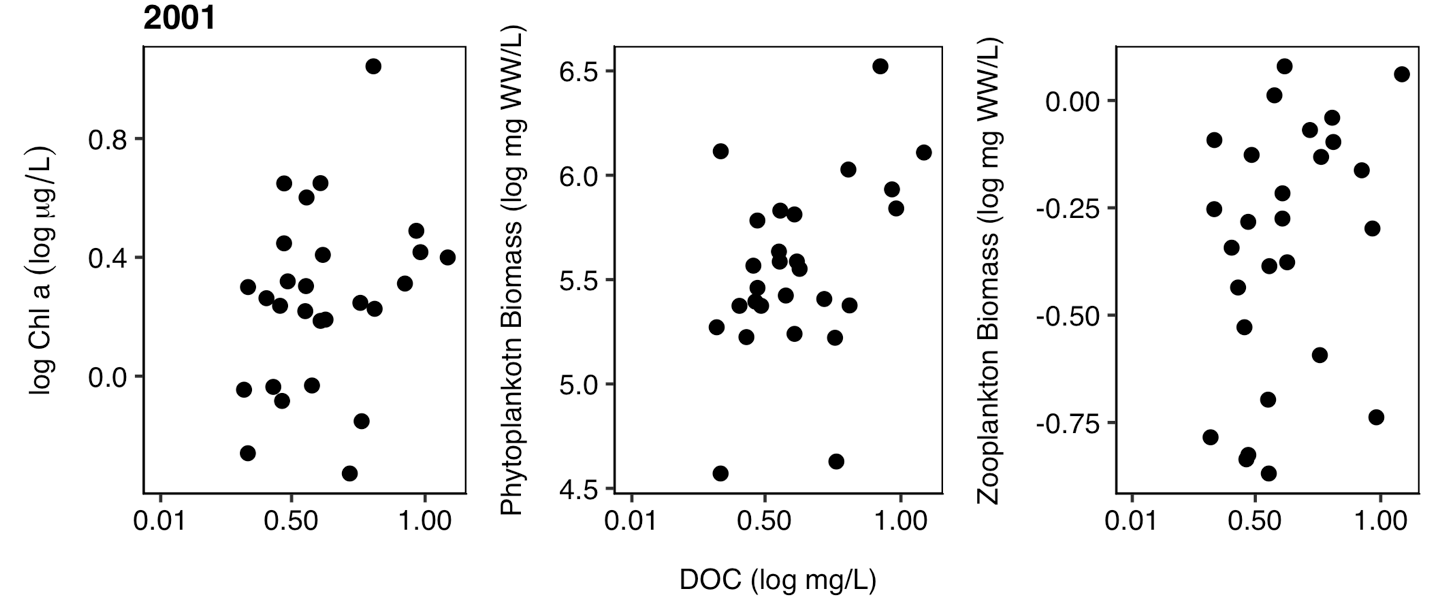

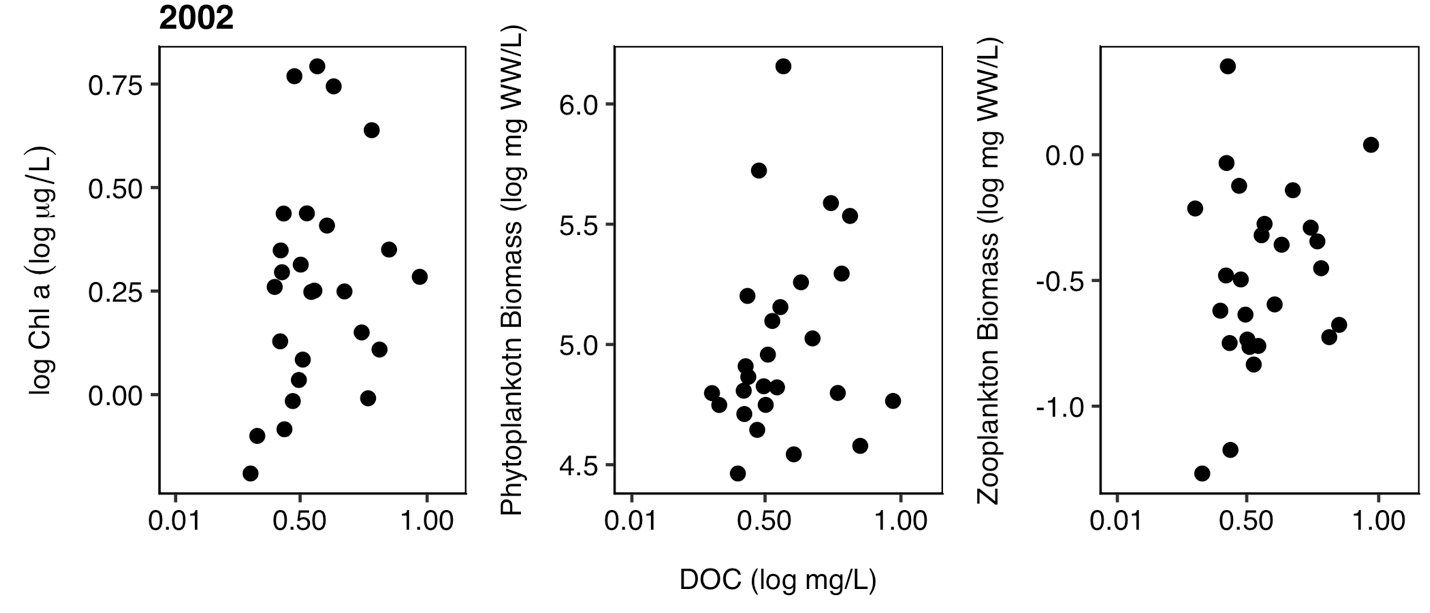

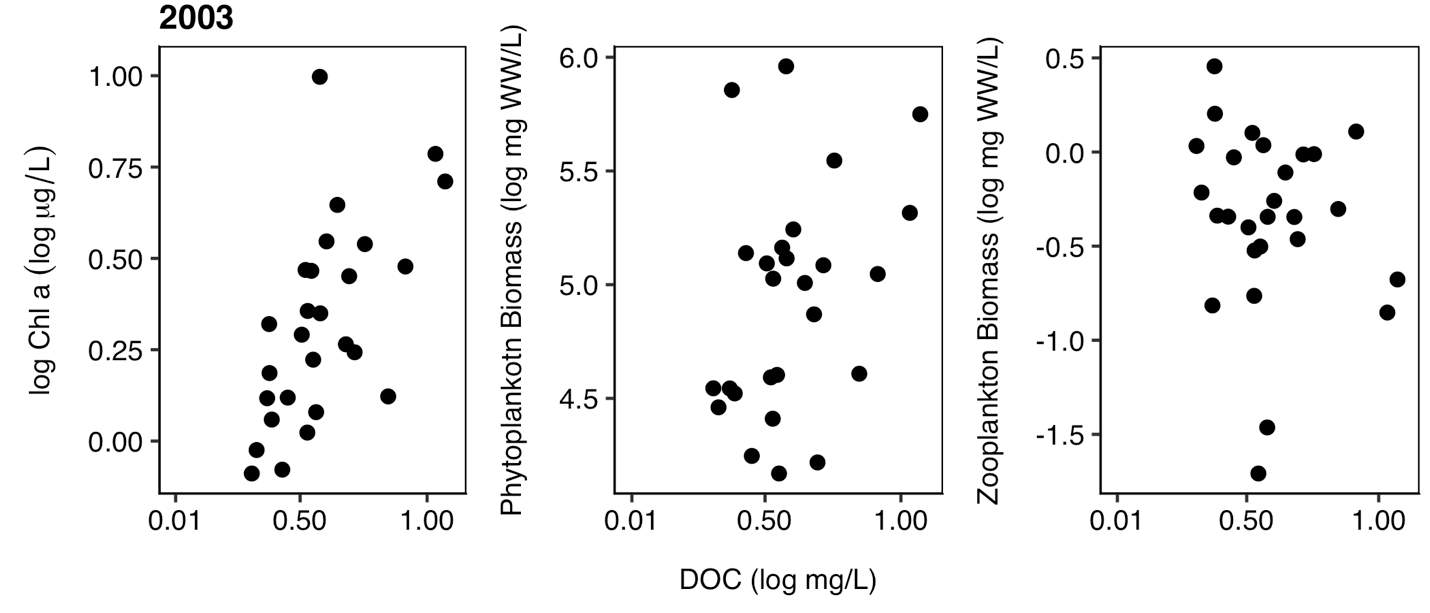

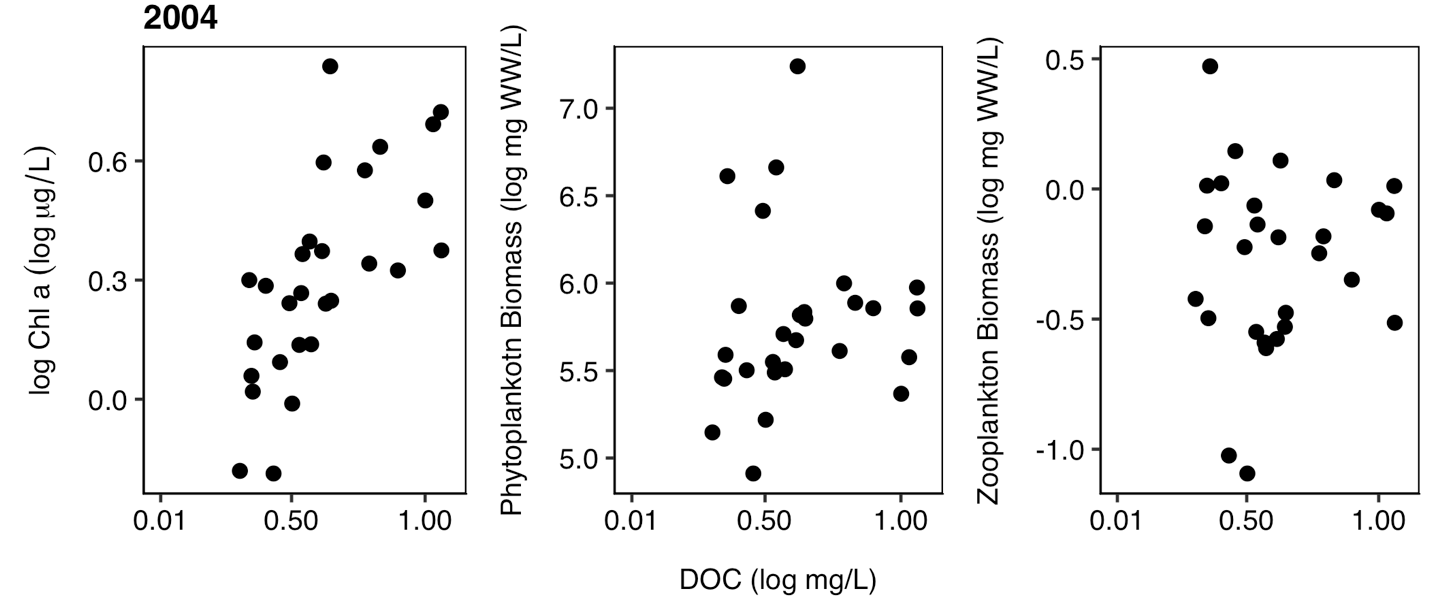

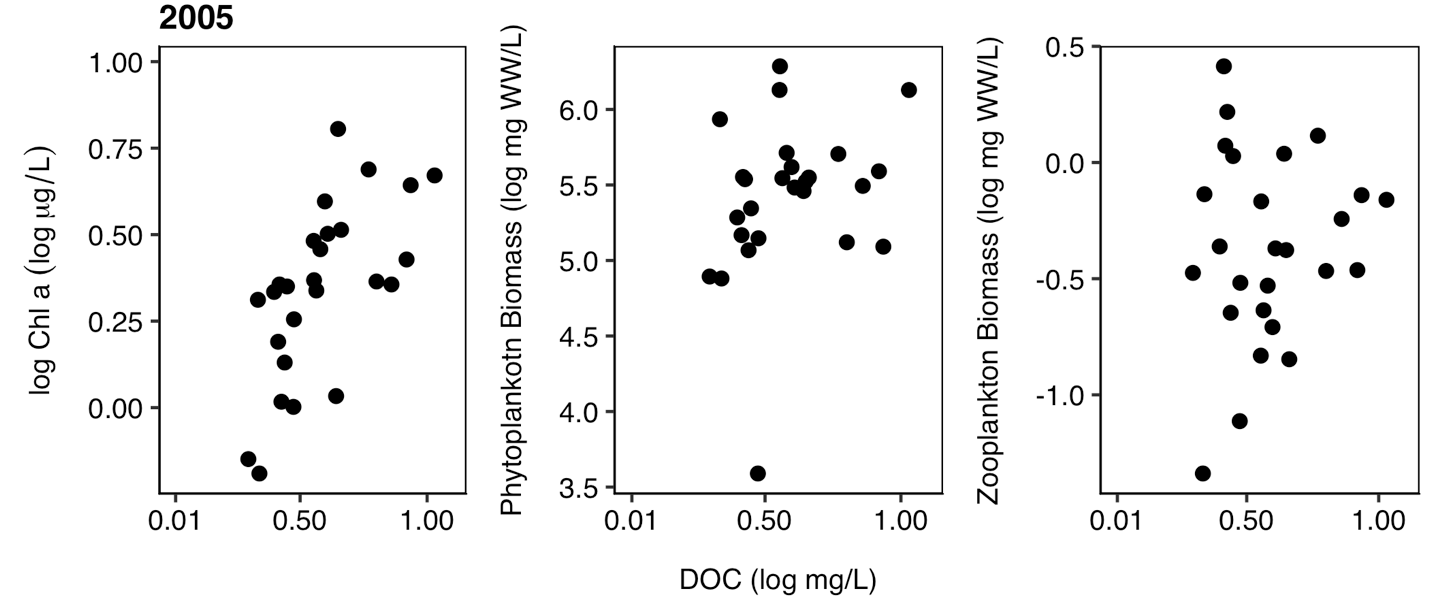

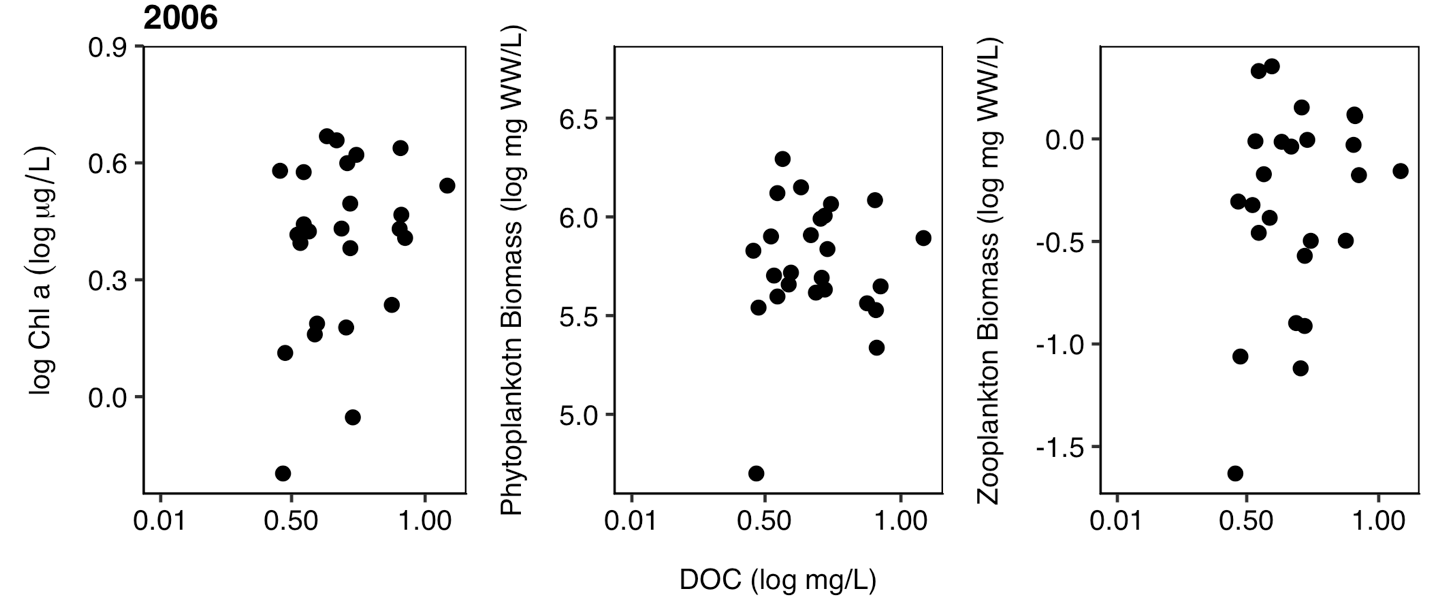


**Table S5**: Model comparison and coefficients for test of unimodal relationship between DOC and chlorophyll, phytoplankton biomass, and zooplankton biomass within a single lake over time. Only lakes with data records from 1994-2012 were considered. All else as in Table S4.

|  | **Linear Model** | | |  | **Quadratic Model** | | | |  |
| --- | --- | --- | --- | --- | --- | --- | --- | --- | --- |
| **Lake** | **β_0_** | **β_1_** | **R^2^** |  | **β_0_** | **β_1_** | **β_2_** | **R^2^** | **∆AIC** |
| ***Chlorophyll*** |  |  |  |  |  |  |  |  |  |
| Big Moose | -0.78 | 1.83 | 0.41* |  | -0.79 | 1.89 | -0.05 | 0.41 | -2.00 |
| Brooktrout | -0.17 | 1.54 | 0.55* |  | -0.20 | 1.11 | 1.10 | 0.57* | -1.27 |
| Cascade | -0.51 | 1.58 | 0.29 |  | -3.29 | 13.15 | -11.84 | 0.39 | 0.69 |
| Dart | -0.51 | 1.27 | 0.67* |  | -0.08 | -0.25 | 1.33 | 0.67* | -1.64 |
| G | 0.23 | 0.70 | 0.06 |  | -0.12 | 2.43 | -2.05 | 0.06 | -1.94 |
| Indian | 0.34 | -0.05 | <0.01 |  | 1.28 | -2.59 | 1.69 | 0.02 | -1.64 |
| Jockeybush | -0.18 | 0.10 | <0.01 |  | -0.86 | 4.40 | -6.35 | 0.14 | 0.67 |
| Limekiln | -0.77 | 1.82 | 0.26 |  | 0.13 | -2.03 | 4.09 | 0.27 | -1.84 |
| Moss | -0.38 | 1.19 | 0.23 |  | -3.64 | 12.35 | -9.42 | 0.30 | -0.16 |
| North | -0.41 | 1.09 | 0.27 |  | -0.97 | 2.89 | -1.39 | 0.29 | -1.68 |
| Rondaxe | -0.04 | 0.75 | 0.29 |  | -1.81 | 6.81 | -5.10 | 0.41 | 1.22 |
| Sagamore | -1.19 | 1.73 | 0.50* |  | -1.11 | 1.57 | 0.09 | 0.50* | -2.00 |
| South | -0.70 | 2.45 | 0.71* |  | -0.74 | 2.69 | -0.33 | 0.71* | -1.99 |
| Squaw | 0.26 | 0.56 | 0.07 |  | 1.94 | -6.08 | 6.42 | 0.16 | -0.10 |
| ***Phytoplankton biomass*** | | | | | | | | | |
| Big Moose | 3.74 | 2.98 | 0.21 |  | 7.17 | -8.98 | 10.19 | 0.23 | -1.55 |
| Brooktrout | 5.27 | 1.01 | 0.37 |  | 5.31 | 1.63 | -1.60 | 0.43 | -0.20 |
| Cascade | 4.69 | 1.56 | 0.03 |  | 9.12 | -16.82 | 18.80 | 0.06 | -1.46 |
| Dart | 4.11 | 2.29 | 0.18 |  | 9.51 | -17.01 | 16.88 | 0.26 | -0.03 |
| G | 5.15 | 1.49 | 0.06 |  | 4.90 | 2.68 | -1.41 | 0.06 | -1.99 |
| Indian | 5.97 | -0.55 | 0.02 |  | 5.30 | 1.26 | -1.21 | 0.02 | -1.98 |
| Jockeybush | 5.01 | 0.25 | <0.01 |  | 4.33 | 4.54 | -6.34 | 0.01 | -1.82 |
| Limekiln | 4.09 | 2.44 | 0.17 |  | 5.85 | -5.12 | 8.03 | 0.18 | -1.80 |
| Moss | 4.63 | 1.32 | 0.09 |  | 3.67 | 4.62 | -2.78 | 0.09 | -1.96 |
| North | 4.24 | 1.56 | 0.13 |  | 6.44 | -5.50 | 5.47 | 0.17 | -1.05 |
| Rondaxe | 4.64 | 1.44 | 0.09 |  | 4.83 | 0.79 | 0.55 | 0.09 | -2.00 |
| Sagamore | 2.94 | 2.85 | 0.21 |  | 16.33 | -26.50 | 15.92 | 0.32 | 0.65 |
| South | 4.94 | 1.43 | 0.05 |  | 6.28 | -6.36 | 10.78 | 0.07 | -1.58 |
| Squaw | 6.52 | -1.16 | 0.02 |  | 4.66 | 6.20 | -7.11 | 0.03 | -1.84 |
| ***Zooplankton biomass*** | | | | | | | | | |
| Big Moose | 0.20 | -1.21 | 0.15 |  | 3.33 | -12.13 | 9.30 | 0.22 | -0.48 |
| Brooktrout | -1.12 | 0.10 | <0.01 |  | -1.05 | 1.08 | -2.50 | 0.10 | -0.14 |
| Cascade | 0.20 | -0.57 | 0.02 |  | 2.83 | -11.51 | 11.19 | 0.06 | -1.14 |
| Dart | 0.51 | -1.97 | 0.50* |  | -1.10 | 3.78 | -5.03 | 0.53 | -0.91 |
| G | -0.46 | 0.58 | 0.04 |  | 0.29 | -3.06 | 4.29 | 0.05 | -1.75 |
| Indian | 0.41 | -0.66 | 0.10 |  | 1.37 | -3.24 | 1.71 | 0.11 | -1.85 |
| Jockeybush | -0.24 | 0.14 | <0.01 |  | -0.19 | -0.21 | 0.52 | <0.01 | -1.99 |
| Limekiln | 0.75 | -2.36 | 0.27 |  | 3.25 | -13.15 | 11.45 | 0.30 | -1.22 |
| Moss | -0.62 | -0.03 | <0.01 |  | 3.62 | -14.55 | 12.25 | 0.04 | -1.25 |
| North | -0.85 | 0.34 | 0.06 |  | -0.53 | -0.68 | 0.79 | 0.07 | -1.81 |
| Rondaxe | -0.03 | -1.03 | 0.16 |  | 1.25 | -5.41 | 3.69 | 0.17 | -1.63 |
| Sagamore | -0.80 | 0.19 | 0.01 |  | -3.96 | 7.12 | -3.76 | 0.08 | -0.69 |
| South | 0.27 | -1.50 | 0.25 |  | 0.11 | -0.54 | -1.32 | 0.25 | -1.96 |
| Squaw | 0.86 | -1.72 | 0.33 |  | 1.56 | -4.48 | 2.67 | 0.34 | -1.77 |

**Figure S2**: Relationship between DOC and chlorophyll concentration, phytoplankton and zooplankton biomass, for all years within a lake. Only lakes with data from 1994-2012 are shown. All values represent annual means. All values represent annual means and are plotted in log-log space but note that scale of axes differ between lakes. See Table 1 for details on each lake.


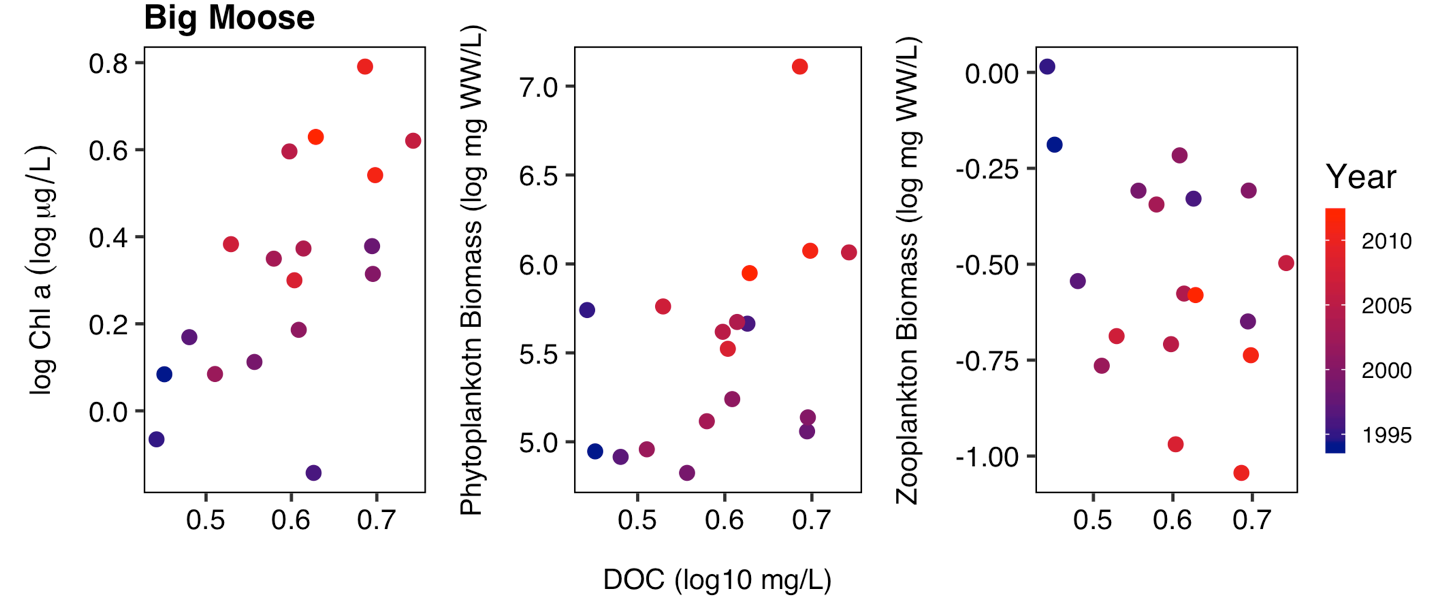


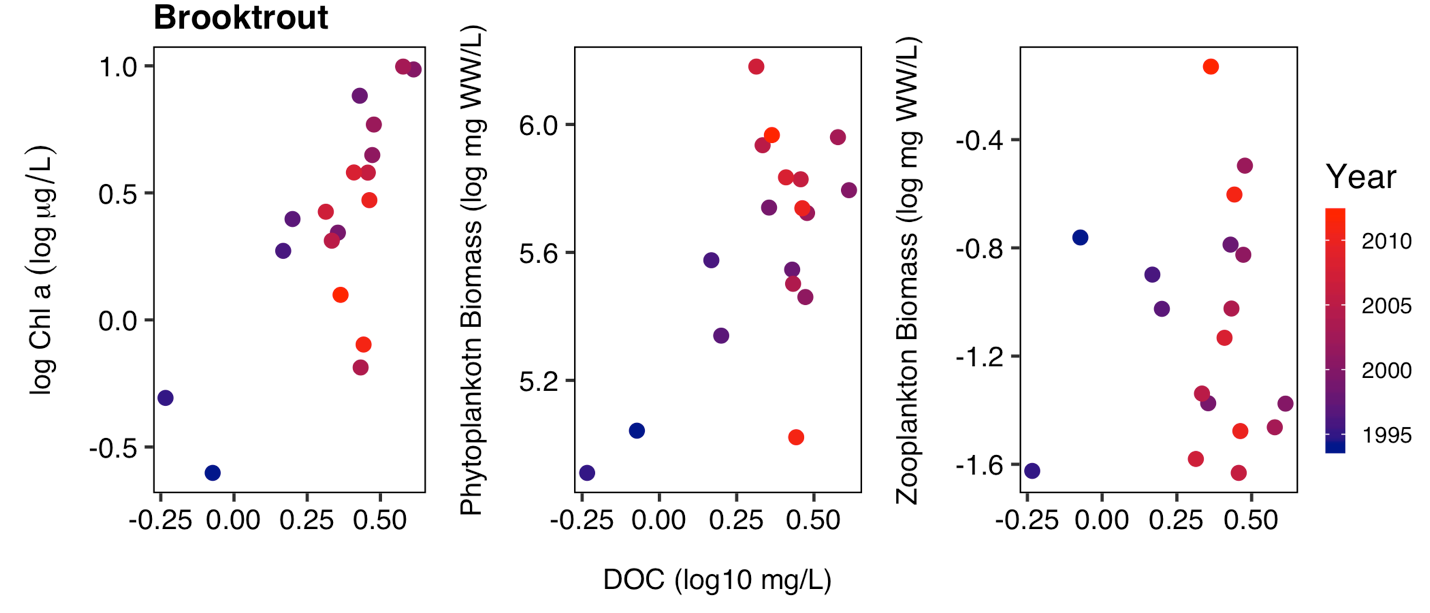


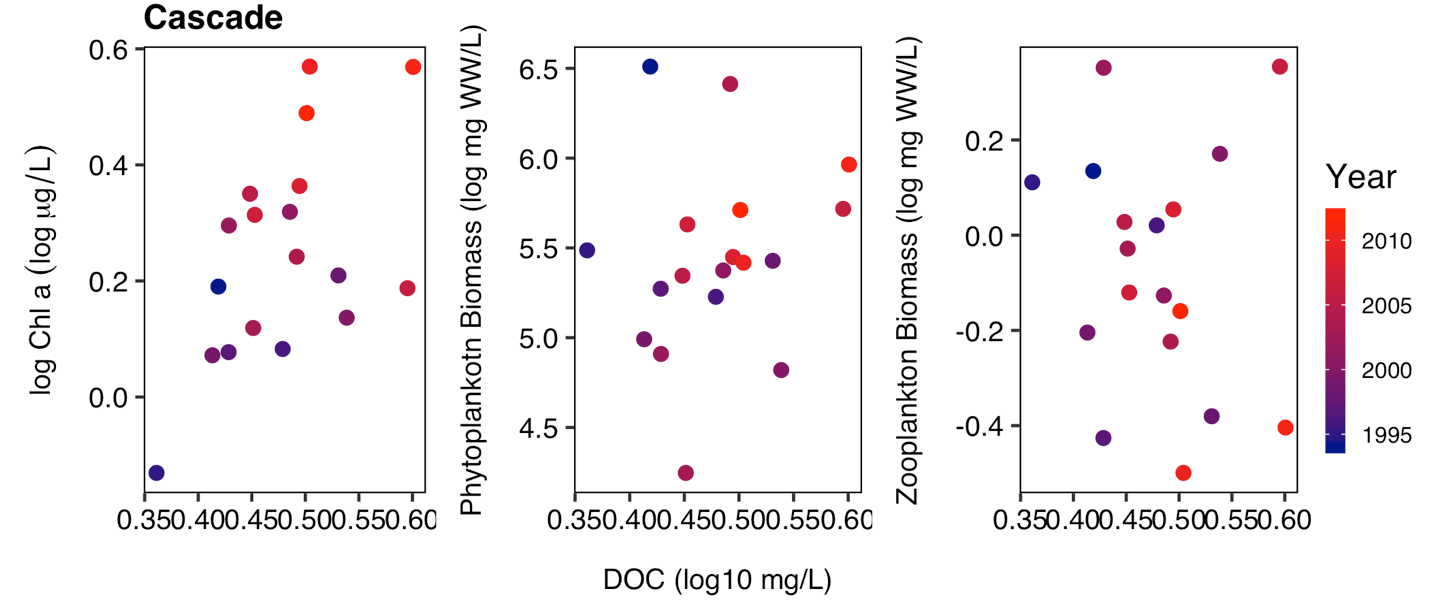

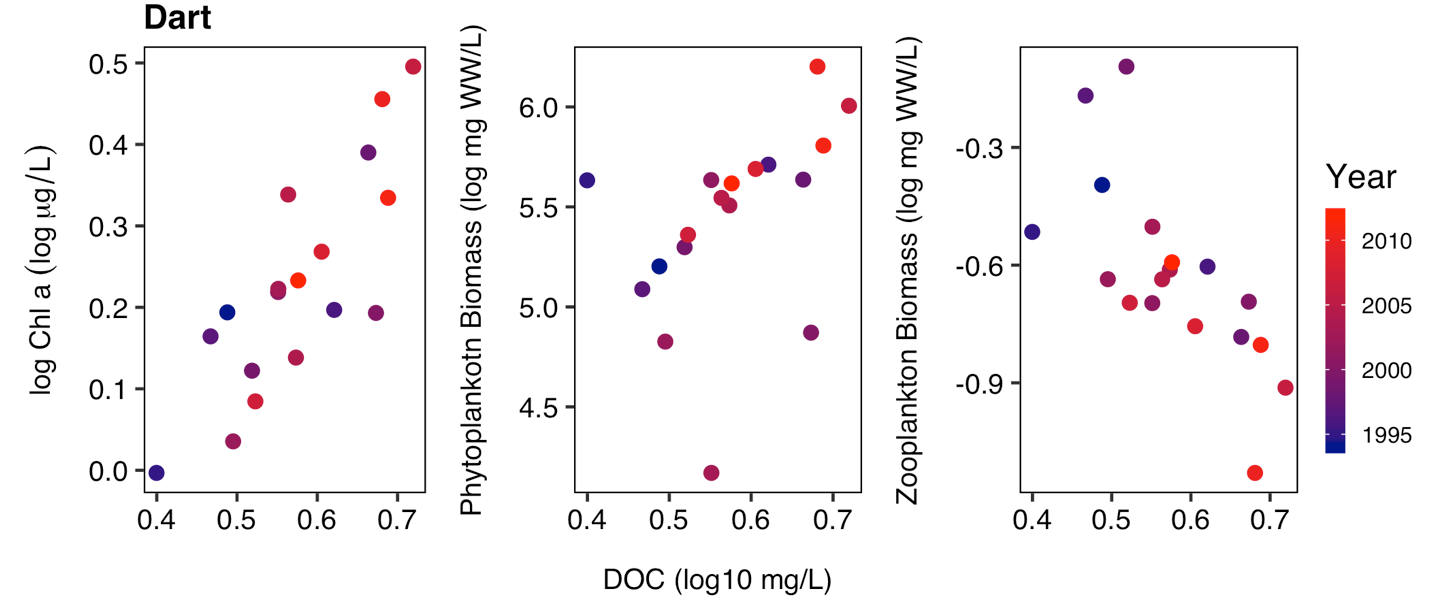

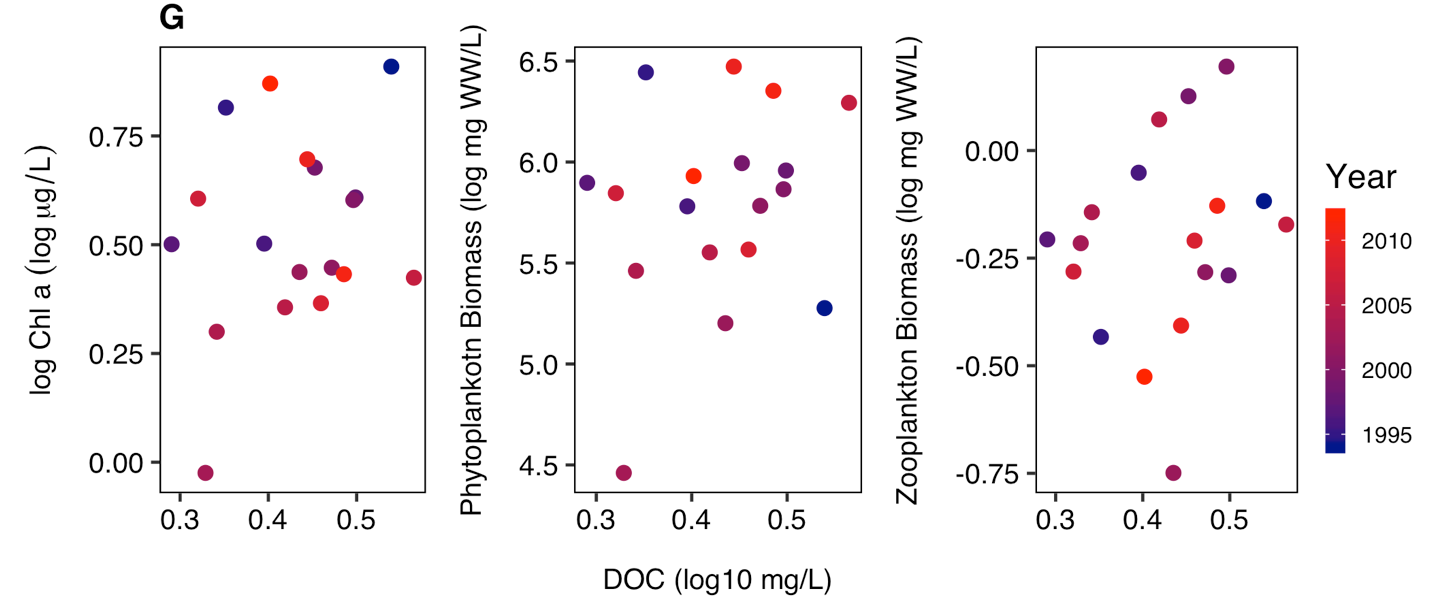


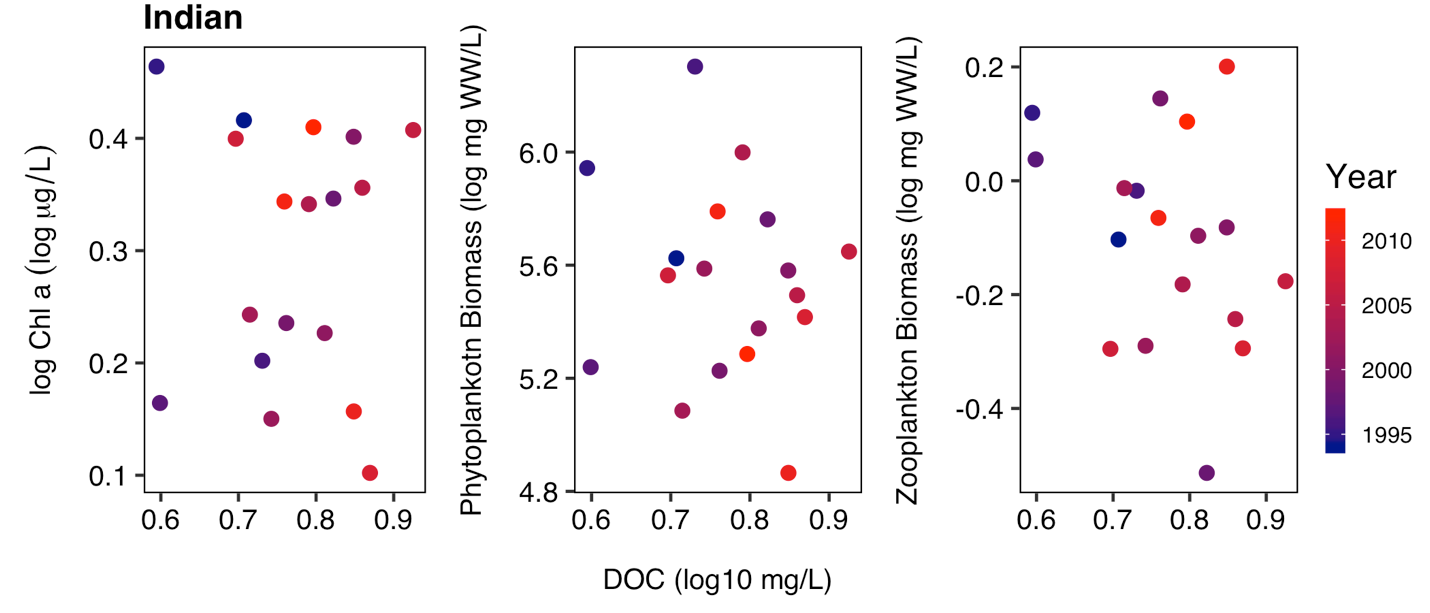

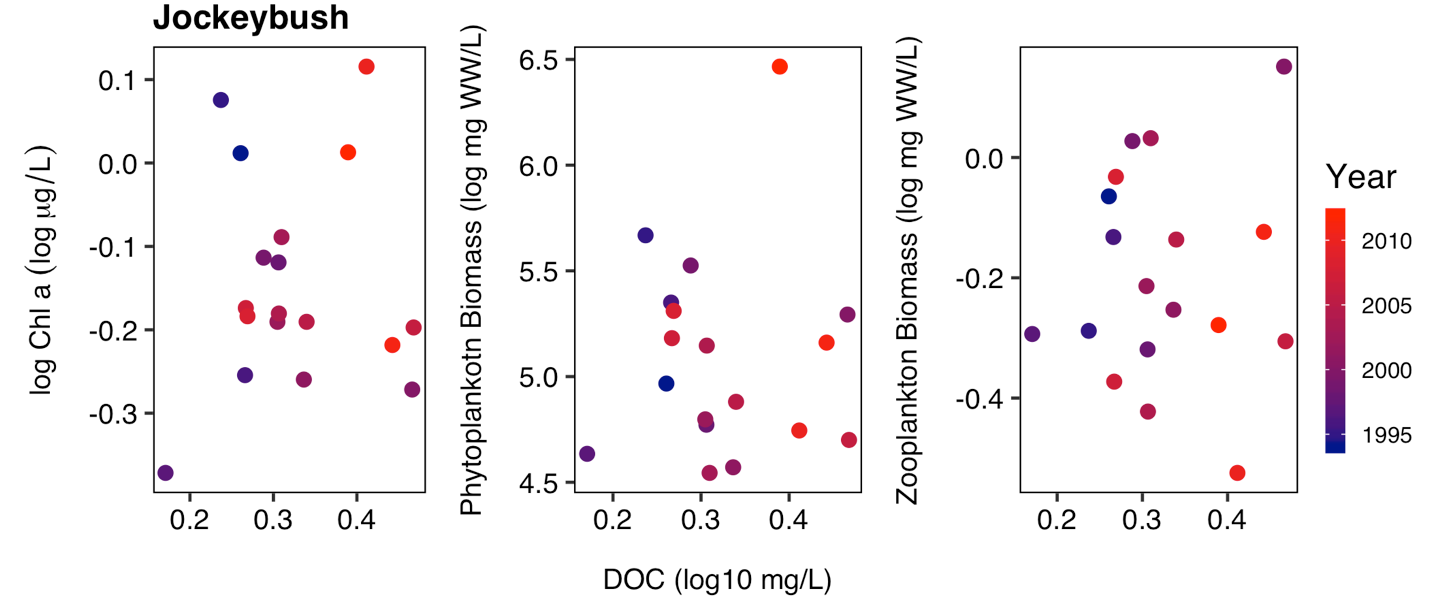

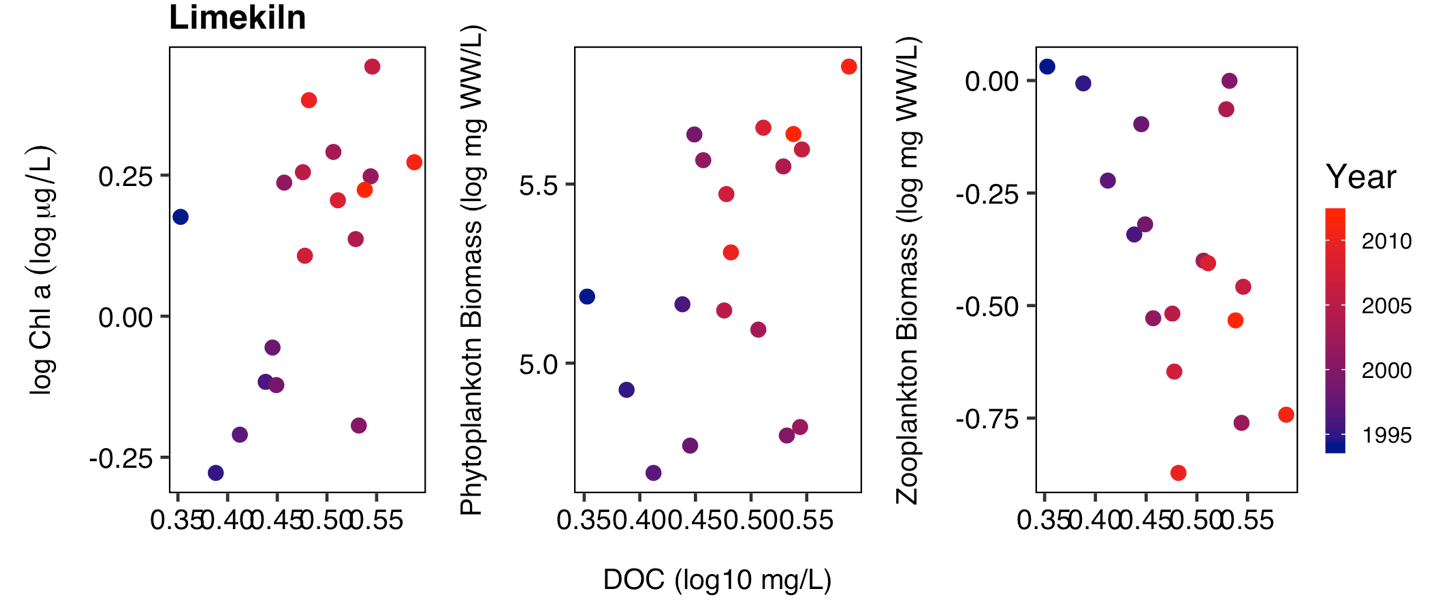


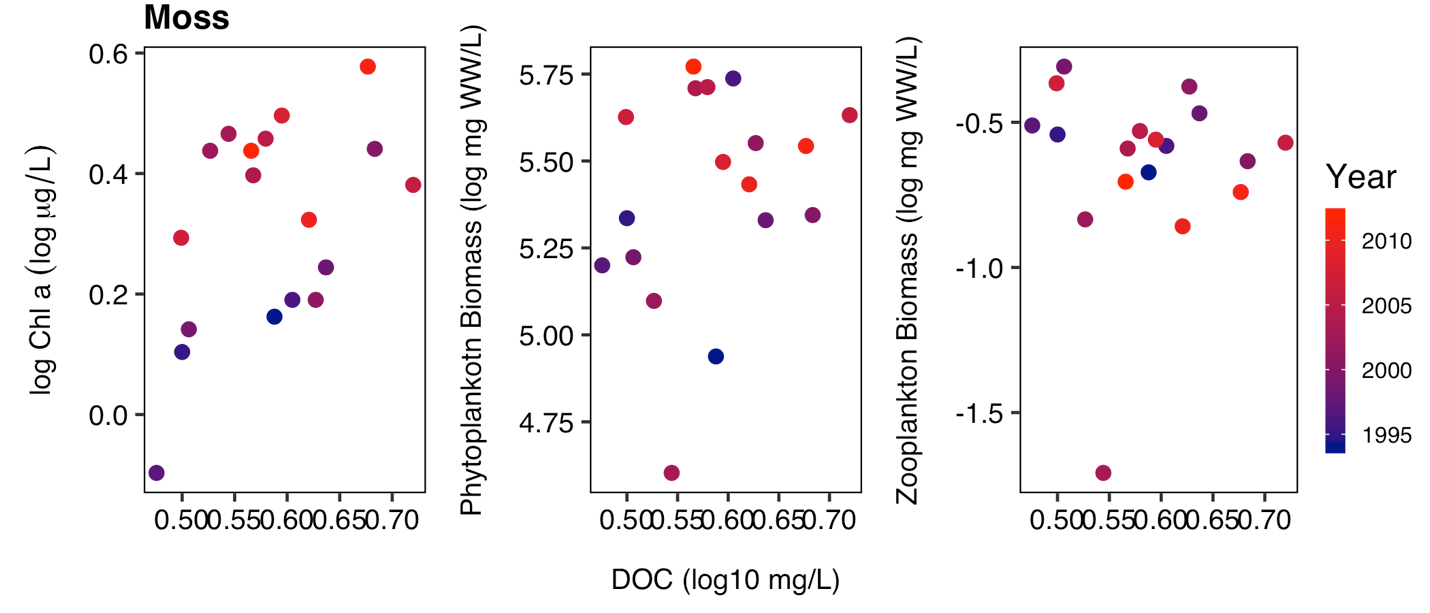

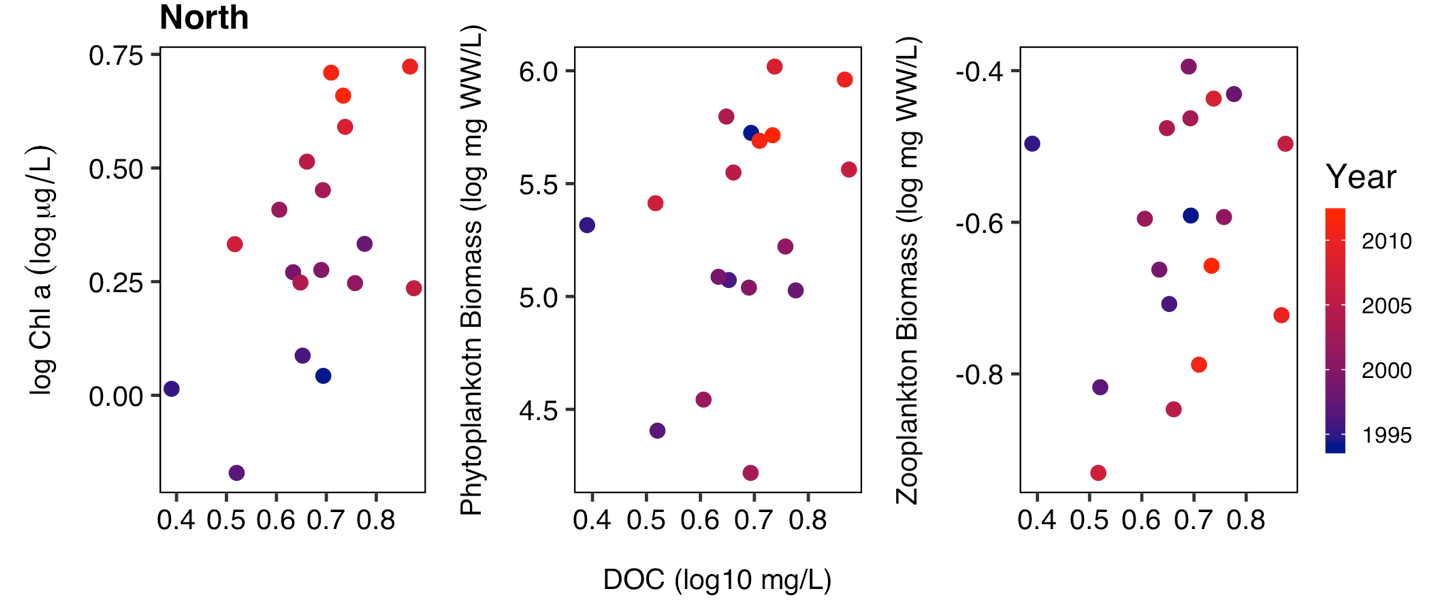

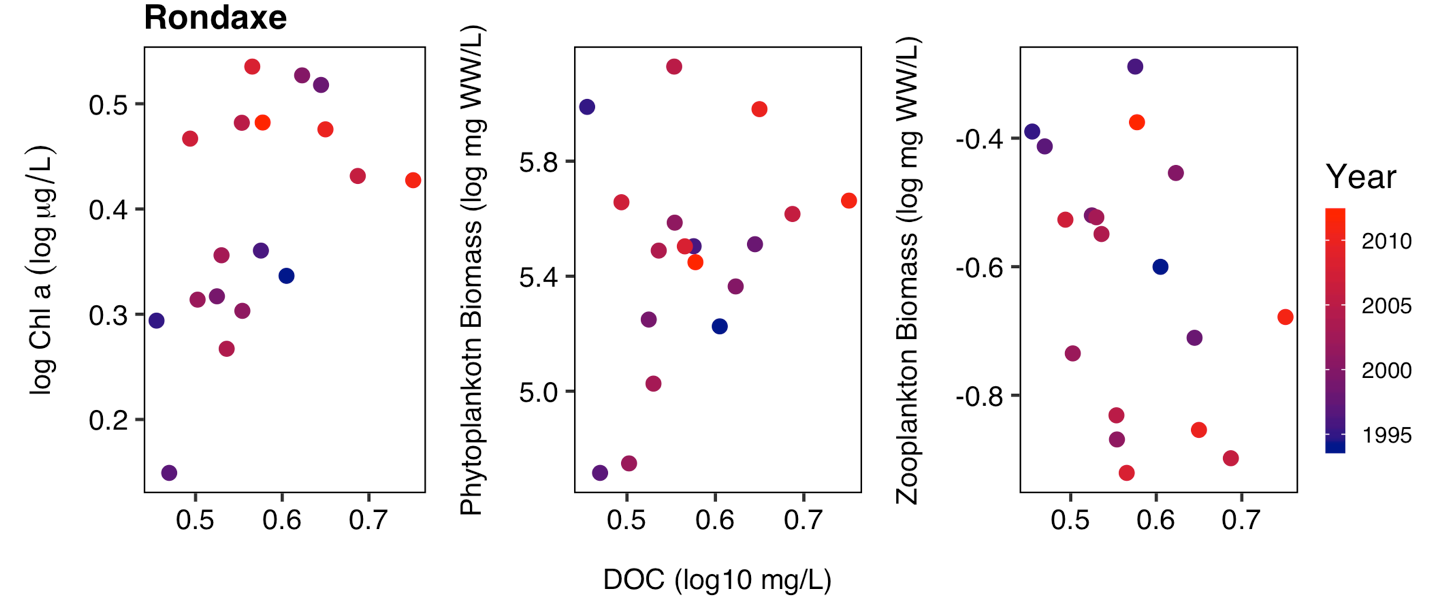

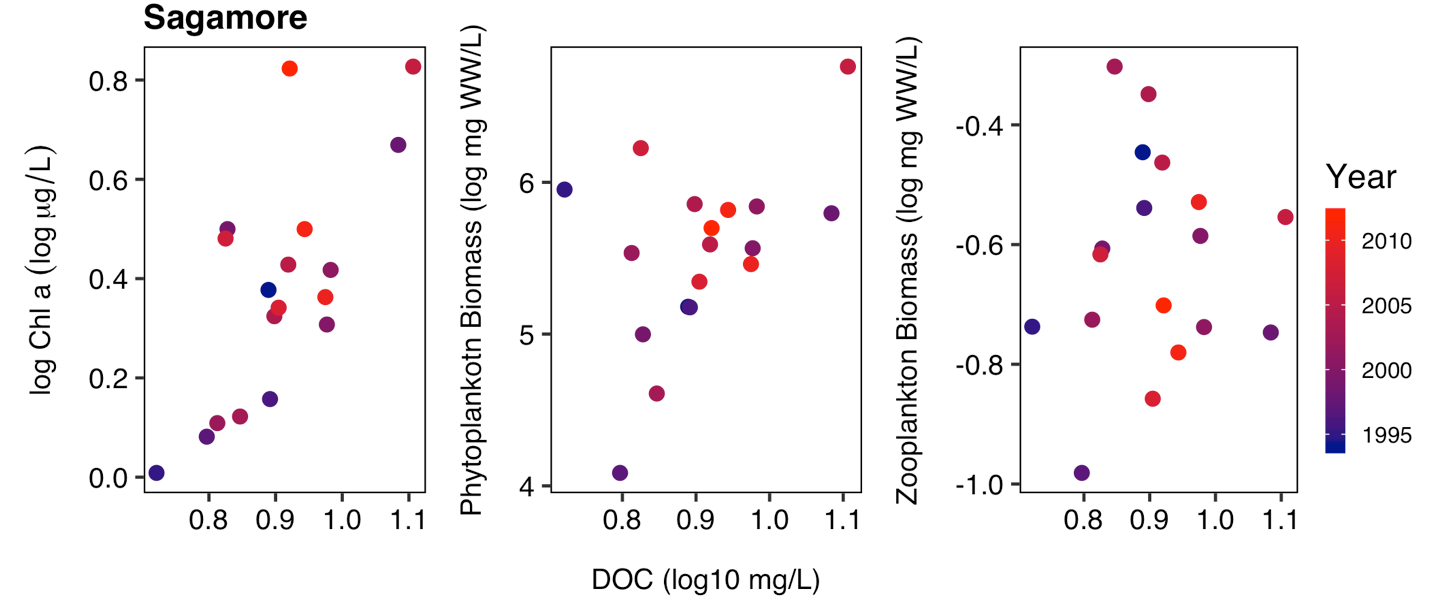

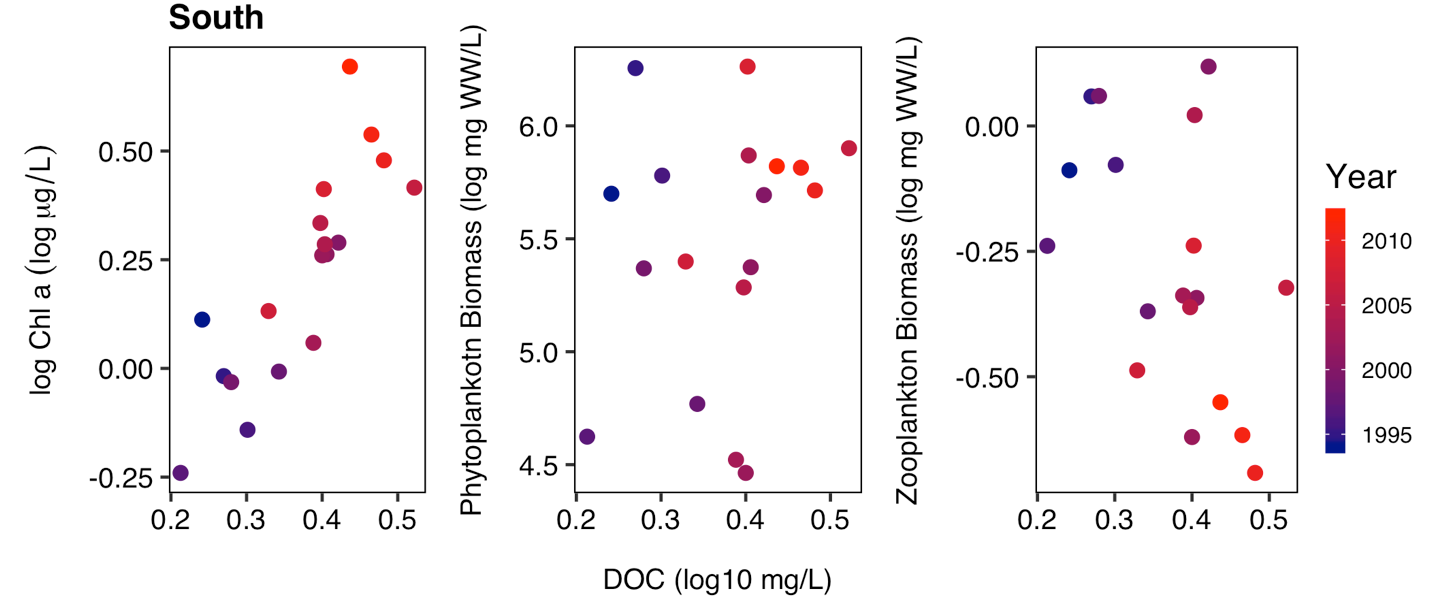

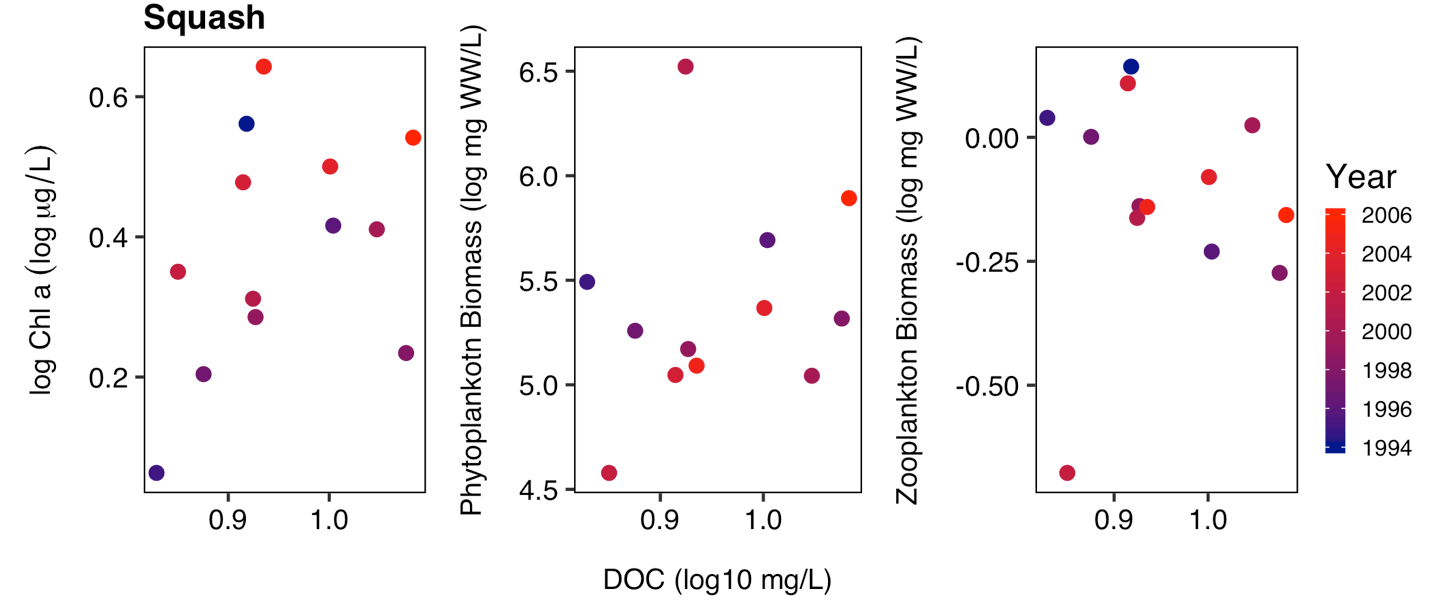

Supplement: Supplementary file 1 [file GCB-25-1779-s001.docx]
